# Role of dermatomes in the determination of therapeutic characteristics of channel acupoints: a similarity-based analysis of data compiled from literature

**DOI:** 10.1186/1749-8546-8-24

**Published:** 2013-12-17

**Authors:** Arthur S Ferreira, Alexandre B Luiz

**Affiliations:** 1 Laboratory of Computational Simulation and Modelling in Rehabilitation, Postgraduate Program of Rehabilitation Science, Centro Universitário Augusto Motta, Praça das Nações 34, 3º andar , Bonsucesso, Rio de Janeiro, Brazil; 2 School of Acupuncture, Centro Universitário Augusto Motta, Rio de Janeiro, Brazil

## Abstract

**Background:**

Analysis of the relationship between the nervous system anatomy and the therapeutic characteristics of all acupuncture points in the channel network may provide new insights on the physiological mechanisms underlying acupuncture stimulation for prevention, treatment, and rehabilitation purposes. This study investigates the association between the similarity of acupoints’ dermatomes, traditional actions, and contemporary indications.

**Methods:**

Channel acupoints had their characteristics annotated from a literature review of four topographic atlases of Chinese medicine and one atlas of human anatomy: initials of the channel’s name (*n* = 14), sequential number in the channel (*n* = 67), acupoint’s name (*n* = 361), dermatomes related to perpendicular needle insertion (*n* = 31), traditional actions (*n* = 848), and contemporary indications (*n* = 1143). Jaccard’s similarity coefficient quantified the similarities between dual acupoints. All dual acupoints were evaluated to generate similarity matrices for each nominal variable. Cross-tables were generated by simultaneous classification of variables into levels of similarity with respect to: dermatomes versus traditional actions, dermatomes *versus* contemporary indications, and traditional actions versus contemporary indications. Goodman-Kruskal γ and Rousson γ*^2^ were calculated based on cross-tables, bootstrap and permutated samples to evaluate the association and determination coefficient between variables, respectively.

**Results:**

Significant associations were observed between levels of similarities of dermatomes and traditional actions (γ = 0.542; *P* < 0.001), dermatomes and contemporary indications (γ = 0.657; *P* < 0.001), and traditional actions and contemporary indications (γ = 0.716; *P* < 0.001). Similarities of dermatomes explained 16% of the variance of traditional actions and 25% of contemporary indications. Traditional actions explained 30% of the variance of contemporary indications. The association between traditional actions and contemporary indications was the highest one (γ = 0.716, 95% confidence interval (95% CI) = [0.715; 0.719]), followed by the association between dermatomes and contemporary indications (γ = 0.622, 95% CI = [0.621; 0.623]), and between dermatomes and traditional actions (γ = 0.446, 95% CI = [0.444; 0.447]), all with *P* < 0.001.

**Conclusions:**

The similarity of dermatomes between dual acupoints partially determined the similarity of traditional actions and contemporary indications, therefore dermatomes partially determine the therapeutic efficacy of acupuncture.

## Background

### Context

Chinese medicine is one of several traditional medical systems practiced by physicians, physiotherapists, nurses, nutritionists, and other healthcare professionals as a coadjutant intervention or even as the single therapeutic intervention [1] for disease prevention, treatment or rehabilitation [2]. Despite of a large amount of research on acupuncture with specific applications such as acupuncture-induced analgesia [3], the biomedical mechanisms related to its therapeutic efficacy remain unclear for many morbid conditions [4].

In Chinese medicine (CM) theory, acupoints are defined as skin loci where vital substances *qi* and *xue* can be manipulated [5]. A philosophy-guided, exploratory analysis of the body surface area (BSA) with consequent systematization of the *jingmai* [channel network] was proposed [5]. The number of acupoints rapidly increased from 160 on the Yellow Emperor’s Inner Classic (*Han* dynasty, 206 BC-220 AD) to 349 on the Systematic Classic of Acupuncture and Moxibustion (*Jin* dynasty, 265–420) [5]. The 361 acupoints used in clinical practice today [6] were already described on the Acupuncture and Moxibustion Feng-Yuan (*Qing* dynasty, 1644–1911). As a consequence, an increase in the number of acupoints results in a ‘high-density mesh’ of acupoints for a given body surface area (BSA), characterized by a decreased inter-acupoint distance – not only on the same channel but also between adjacent ones.

Both scientific definition and anatomical substrate of acupoint remains debatable [7-16]. The correspondence to a dermatome pattern [7], the presence of neurovascular bundles [8,9], different types of terminal nerves [9-11], and a reduced skin electric impedance [12,13] are amongst the most common characteristics attributed to acupoints [14,15]. The material basis of acupuncture is the nerves since the nervous system is the common factor between many scientific evidence on acupuncture’s efficacy [16].

A dermatome refers to a cutaneous area innervated by one nerve element, specifically nerve root, dorsal ganglion or spinal segment [17]. Either inconsistent or incomplete results were obtained by several studies. A study on the segmental innervation of acupoints observed that the needle stimulation of ‘correct’ skin loci for acupoints *Yanglingquan* GB34, *Yinlingquan* SP9, and *Xiangu* S43 resulted in zones of hyposensitivity confirming to a dermatome pattern of L3 and L5 [7]. Mayor [18] found no justification based on dermatomes for selecting the *shu* [traditional transporting] and *mu* [alarm] acupoints for an organ rather than some other *shu* or *mu* acupoints associated with another organ. Cabioglu *et al.*[19,20] investigated both *shu* and *Huato-Jiaji* acupoints and *mu* acupoints and suggested that the application of acupuncture might balance sympathetic and parasympathetic activities. Cheng [21] suggested that in ‘many cases’ the acupoints have a neuroanatomical relationship to Western medicine. Sánchez-Araujo *et al.*[22] proposed a distinction between a ‘meridian-derived’ and a ‘neurobiology-derived’ effects depending on whether the therapeutic effects of acupoints were related to the corresponding *zangfu* [internal organs] or dermatome, respectively. These authors performed a partial literature compilation and found that acupoints effects for any dermatome are remarkably similar regardless of their corresponding channel [22]. Recently, Silva [23] suggested minor corrections to the optimal dermatomes of *shu* acupoints to stimulate the internal organ function.

Most of those studies were limited to one or few acupoints located in one or few body parts or channels, and extrapolation of the results to all channels’ network might not be not accurate. Furthermore, the applied methods only investigated the anatomical substrate of acupoints but not the relationship between anatomical and therapeutic characteristics attributed to acupoints.

Recurrent utilization of acupoints by many CM scholars led to the discovery and establishment of acupoints’ traditional actions, which were combined to their previous known ones. Likewise, clinical practice and scientific research led to a new set of contemporary indications of acupoints [5]. Scientific evidence suggested that channel-based therapies could treat several morbid conditions [1,4], but acupoints’ specificity regarding treatment have been questioned [24,25]. Clinical studies showed either a large variability in acupoint selection for the same clinical condition [26] or similar effectiveness between sham and verum acupuncture [1,27]. Additionally, a recent review considered the specificity of acupoints as controversial despite some scientific evidence [28]. Although acupoints were commonly considered as small cutaneous region, it was found a large variance on the skin surface area for the exact location of acupoints by CM experts [29]. This large variation may stimulate different skin loci – and possibly different dermatomes inducing unexpected or new therapeutic effects. On the contrary, the high-density mesh of acupoints in the channel network may result in the stimulation of common dermatomes for acupoints lying ‘close enough’ to each other, inducing therapeutic characteristics similar to nearby acupoints.

This study aims to investigate the association between the similarity of acupoints with respect to their dermatomes, traditional actions, and contemporary indications by a similarity-based analysis of their literature content. It was hypothesized that the strength of similarity of dermatomes of dual acupoints determines the strength of their similarity concerning either traditional actions or contemporary indications, suggesting a role of dermatomes for determination of the therapeutic characteristics of channel acupoints.

## Methods

### Study design and assumptions

**Figure 1:**
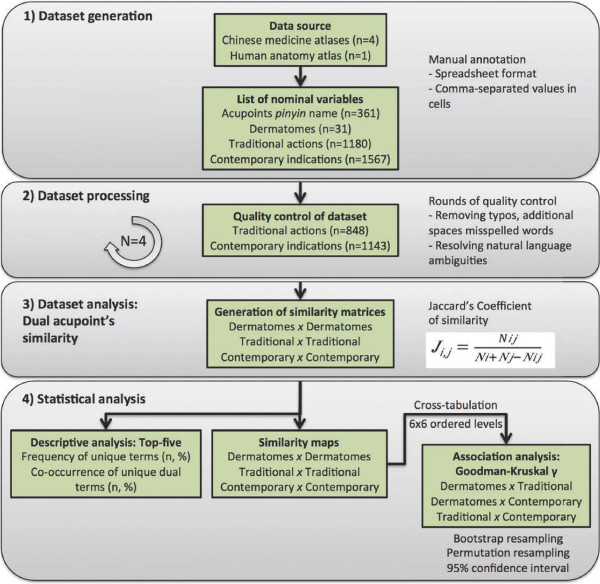
**Flowchart of the study.** The flow chart of the study was divided into stages: dataset generation, dataset quality control, computation of dual acupoint’s similarity, and statistical analysis.

This literature review study was divided into dataset generation, processing, and analyses (Figure 1). Information from acupoints was compiled from four topographic atlases of CM acupoints [30-33] and one atlas of human anatomy [34]. Dermatomes related to acupoints were obtained from only one of those atlases [30] because of its systematic description of the anatomy of needle insertion. Traditional actions and contemporary indications of acupoints were firstly annotated from the same atlas [30] and completed with information from the other three atlases [31-33]. The anatomical descriptions on those atlases were verified with a human anatomy textbook [34], chosen due to its systematic description.

In this study, we assumed that (1st) the traditional actions of acupoints described in the literature reflected the state-of-the-art knowledge on acupoints; (2nd) the contemporary indications of acupoints described in the literature reflected the state-of-the-art knowledge on acupoints; (3rd) the dermatome information in these atlases was accurately described.

### Computational resources

The complete dataset of acupoints was shown (Additional file 1, in Portuguese language). Algorithms for dataset processing, dataset analysis and statistical analysis were implemented in Maple 15 (Waterloo Maple Inc., USA) (Additional file 2). The corresponding worksheet in editable mode was available (Additional file 3). All algorithms were executed on a 2.26 GHz Intel® Core 2 Duo microprocessor with 2 GB RAM running Mac OS X 10.8.2 (Apple Inc., USA).

### Dataset generation

Acupoints had the following characteristics annotated as nominal variables in the raw dataset: capitalized initials of the channel’s name (*n* = 14), sequential number in the channel (*n* = 67), acupoint’s name (*n* = 361), dermatomes related to perpendicular needle insertion (*n* = 31), traditional actions (*n* = 1180), and contemporary indications (*n* = 1567), and other information not used in this study (circulation level, channel’s six-level theory, moxibustion duration, needle insertion depth, and myotome). The first three variables were concatenated to generate full and unique identification of acupoints, *e.g.* CV21 *Xuanji*. Deviations from the anatomical norm on dermatomes as well as missing data regarding local innervation on the CM atlases were checked by the human anatomy atlas [34]. Nominal variables that present with one or more values per acupoint (dermatomes, traditional actions, and contemporary indications) were annotated as a single text entry formatted with comma separated values (CSV).

### Dataset processing: quality control of the channel acupoints’ dataset

The natural language in the consulted literature may exhibit ambiguities in terms concerning traditional actions and contemporary indications of acupoints, such as plurals, synonyms, capitalized words, and use of different prepositions. Therefore, the raw dataset passed a two-steps procedure to produce a list with a controlled vocabulary to ensure high-quality data before statistical analysis:

(1st) All typos due to manual annotation were corrected by the worksheet text processor dictionary. Misspelled terms were corrected and additional spaces before comma, between words, and after commas and words were removed. Capitalized words were converted to lowercase ones.

(2nd) Each data column containing traditional actions and contemporary indications – from all acupoints was firstly merged and then separated into individual terms for generating two independent lists of unique terms. Both lists were simultaneously inspected by two observers (ABL and ASF) for repetition of terms and ambiguities were minimized as follows. Synonymous were replaced by the same exact term because they represent the same therapeutic action (*e.g.* “strengthens the lumbosacral region” and “strengthens the lumbar region” were replaced by the latter term). Composite terms were split into individual terms because they comprised different therapeutic actions that co-occur (*e.g.* “tonifies *qi* and *xue*” was replaced by “tonifies *qi*, tonifies *xue*”). This second step was repeated until both observers did not change either lists, counting up four rounds of dataset processing.

All changes per round were indicated in the Additional file 1, as well as the final list of terms. After this quality control procedure, the quantities of unique terms describing the traditional actions and contemporary indications in the processed dataset were reduced by 28% (*n* = 848) and 27% (*n* = 1143), respectively.

### Dataset analysis: computation of dual acupoints’ similarity

Skin loci of acupoints were usually supplied by two or more dermatomes because of superimposition of cutaneous innervation, and thus acupoints shared dermatomes depending on whether or not they were close to each other in the channel network. Likewise, both traditional actions and contemporary indications were sets of terms describing the expected therapeutic usefulness of acupoints, which might also be shared with other acupoints either close enough or apart each other due to their local or systemic effects, respectively [5]. Therefore, a measure of co-occurrence of terms was selected to quantify similarities between dual acupoints based on these nominal variables. For each dual acupoints *i* and *j*, the Jaccard’s similarity coefficient *J*_*i, j*_[35] was calculated as following:

(1) $$J_{i,j}=\frac{N_{i,j}}{N_{i}+N_{j}-N_{i,j}}$$

where *N*_*ij*_ is the number of terms contained in both acupoints, and *N*_*i*_ and *N*_*j*_ are the number of terms contained in either acupoints *i* or *j* (*i* = 1, 2, …, 361 and *j* = 1, 2, …, 361) members of the dual acupoint. Coefficient *J*_*i, j*_ indicates the strength of similarity between acupoints *i* and *j* and ranges from perfect dissimilarity (*J*_*i, j*_ = 0) to perfect similarity (*J*_*i, j*_ = 1). For instance, the lower boundary condition for dermatomes was satisfied by dual acupoints that did not share any dermatomes (perfectly dissimilar dual acupoints), while the upper boundary condition is satisfied by dual acupoints that shared all dermatomes (perfectly similar dual acupoints). The same reasoning applied to traditional actions and contemporary indications as separated variables.

The dataset defined 130,321 dual acupoints in a symmetrical matrix, including the elements at the main diagonal. Similarity matrices were calculated for the following variables considering all dual acupoints: dermatomes (*D*_*361,361*_), traditional actions (*T*_*361,361*_), and contemporary indications (*C*_*361,361*_). Acupoints in all similarity matrices were arranged with the rostral-caudal sequence of dermatomes level: C2, C3, …, S4, S5. Within the same dermatomes the acupoints were arranged in the sequence of *qi* circulation among channels: *du mai* [*Governing vessel*], *ren mai* [*Conception vessel*], *fei* [*Lung*], *dachang* [*Large Intestine*], *wei* [*Stomach*], *pi* [*Spleen*], *xin* [*Heart*], *xiaochang* [*Small Intestine*], *pangguang* [*Bladder*], *shen* [*Kidney*], *xinbao* [*Pericardium*], *sanjiao* [*Triple Burner*], *dan* [*Gallbladder*], and *gan* [*Liver*] [5]. This ‘dermatome/channel’ arrangement allowed the selection of corresponding pairs of dual acupoints’ similarity in two different matrices by the same indexing.

### Statistical analysis

Literature compilation was characterized by descriptive analysis using absolute and relative frequencies. The top-five most frequent traditional actions and contemporary indications were identified. Also, top-five co-occurrence of terms in dataset were described for the following pairs: (1) traditional versus traditional actions; (2) traditional actions versus contemporary indications; and (3) contemporary versus contemporary indications.

Similarity maps were generated from matrices *D*, *T*, and *C* in dermatome/channel arrangement and after shuffling to visualize the effect of sequence on the similarity map. Grey-scaled mapping was used to indicate low (= blackish) and high (= whitish) values of similarity, respectively.

Cross-tables were generated from similarity matrices with respect to: (a) dermatomes versus traditional actions (*D*_*i, j*_*x T*_*i, j*_); (b) dermatomes versus contemporary indications (*D*_*i, j*_*x C*_*i, j*_); and (c) traditional actions versus contemporary indications (*T*_*i, j*_*x C*_*i, j*_). Each cross-table was established by simultaneous classification of all *J*_*i, j*_ values from the two matrices into the following categories representing the strength of similarity:

(1st) 0.000 |— 0.167: Null or negligible;

(2nd) 0.167 |— 0.333: Very weak;

(3rd) 0.333 |— 0.500: Weak;

(4th) 0.555 |— 0.667: Moderate;

(5th) 0.667 |— 0.833: Strong;

(6th) 0.833 |— 1.000: Very strong.

Perfect similarity was not included in the last class because acupoints with *J*_*i, j*_ = 1 were found only in the main diagonal of matrices. Goodman-Kruskal γ [36] was calculated from the cross-tables to evaluate the monotonic linear-to-linear association between the strength of dual acupoint similarities. The squared value of the Rousson γ* (a variant of Goodman-Kruskal γ [37]) was calculated to represent the coefficient of determination between the ordinal variables, *i.e.* the percentage of variation in one variable that was explained by the other variable.

Statistical significance of Goodman-Kruskal γ were obtained based on the analysis of 95% confidence intervals (95% CI) estimated by bootstrap resampling with *B* = 1000 replications of dual acupoints on the similarity matrices *D*_*i, j*_*x T*_*i, j*_, *D*_*i, j*_*x C*_*i, j*_, and *T*_*i, j*_*x C*_*i, j*_. Median and confidence intervals for the Goodman-Kruskal γ and Rousson γ*^2^ were estimated based on the percentile method (2.5th and 97.5th) of the bootstrap samples [38,39]. Permutation test was also performed by bootstrap resampling with *B* = 1000 replications of different pairs of acupoints in the similarity matrices *D*_*i, j*_*x T*_*k, q*_, *D*_*i, j*_*x C*_*k, q*_, and *T*_*i, j*_*x C*_*k, q*_ to test the null hypothesis that there was no linear association (γ = 0; *P* ≥ 0.05) between each tested pairs of variables classified by their levels of similarity. *P* values were calculated as the proportion of the Goodman-Kruskal γ values generated in the permutation test that was larger than the Goodman-Kruskal γ calculated from the original cross-tables [39].

## Results

### Example acupoints

In the following examples, the texts in italic format represented terms shared between acupoints. Considered the CV21 *Xuanji* (*i* = 49) and CV22 *Tiantu* (*j* = 50) as a first dual acupoint, they were described in dataset by dermatomes: CV21 = {*C3*, C4} and CV22 = {C2, *C3*}. Their respective traditional indications were: CV21 = {regulates the *qi*, *alleviates the asthma***,***alleviates the cough*, harmonizes the thoracic *qi*, harmonizes the *qi* inversion} and CV22 = {tonifies *fei-qi*, dominates *qi* inversion, promotes *fei-qi* circulation**,***alleviates the asthma*, eliminates heat, remove humidity, befits the throat, refreshes the throat, clear the voice, stops the cough}. Also, their contemporary indications were: CV21 = {*asthma*, *chronic bronchitis*, tonsillitis, pulmonary emphysema, pleurisy, spasm of the oesophagus, spasm of the stomach, *difficult deglutition*, *phlegm*, *aphony*} and CV22 = {*asthma*, *chronic bronchitis*, acute bronchitis, pharyngitis, hiccup, acute cough, chronic cough, *aphony*, *phlegm*, sudden dysphonia, *difficult deglutition*, goitre, pertussis, reflux, vocal cord diseases, sudden snoring}. Therefore, the calculated Jaccard’s similarities were *D*_*49,50*_ = 0.33, *T*_*49,50*_ = 0.07, and *C*_*49,50*_ = 0.24.

Considered another dual acupoint with the same acupoint CV21 *Xuanji* (*i* = 49) but with LU6 *Kongzui* (*j* = 58). Acupoint LU6 was described by dermatomes LU6 = {C5, C6}, its traditional indications were LU6 = {reduces fever, descends *fei-qi*, regulates *fei-qi*, stops haemorrhage, *alleviates the cough***,** alleviates the dyspnoea}, and its contemporary indications were LU6 = {headache, dysphonia, cough, throat pain, elbow pain, fever, tuberculosis, acute asthmatic crisis, epistaxis}. Therefore, the calculated Jaccard’s similarities were *D*_*49,58*_ = 0.00, *T*_*49,58*_ = 0.10, and *C*_*49,58*_ = 0.00.

### Descriptive analysis of literature compilation

Both frequencies and co-occurrences of terms were presented in Table 1. The most frequent traditional action was “dispels *wind*” (*n* = 140, 38.8%) and the most frequent contemporary indication was “headache” (*n* = 100, 27.7%). Co-occurrences of traditional versus traditional actions, traditional actions versus contemporary indications, and contemporary versus contemporary indications represented *n* = 11579, *n* = 42928, and *n* = 39039 unique dual terms, respectively. Dual terms “dispels *wind*–dispels *heat*” (*n* = 77, 21.3%), “dispels *wind*–headache” (*n* = 69, 19.1%), and “asthma–cough” (*n* = 49, 13.6%) were the most frequent ones, respectively.

**Table 1:** Descriptive analysis of terms from the literature compilation

**Category**	**Terms**	**N**	**%**
**Traditional actions**	(1) Dispels *wind*	140	38.8
*N* = 848 terms (no repetition)	(2) Dispels *heat*	136	37.7
	(3) Reduces fever	90	24.9
	(4) Calms the mind	81	22.4
	(5) Eliminates pain	68	18.8
**Contemporary indications**	(1) Headache	100	27.7
*N* = 1143 terms (no repetition)	(2) Low back pain	95	26.3
	(3) Asthma	78	21.6
	(4) Vomiting	73	20.2
	(5) Convulsion	71	19.7
**Traditional *vs. *** **traditional**	(1) Dispels *wind* – dispels *heat*	77	21.3
*N* = 11579 pairs of terms (no repetition)	(2) Dispels *wind* – eliminates pain	48	13.3
	(3) Dispels *wind* – reduces fever	47	13.0
	(4) Dispels *damp* – dispels *heat*	40	11.1
	(5) Calms the mind – dispels *heat*	36	10.0
**Traditional *vs. *** **contemporary**	(1) Dispels *wind* – headache	69	19.1
*N* = 42928 pairs of terms (no repetitions)	(2) Dispels *heat* – headache	60	16.6
	(3) Dispels *wind* – vertigo	45	12.5
	(4) Calms the mind – epilepsy	43	11.9
	(5) Dispels *wind* – convulsion	42	11.6
**Contemporary *vs. *** **contemporary**	(1) Asthma – cough	49	13.6
*N* = 39039 pairs of terms (no repetition)	(2) Convulsion – epilepsy	45	12.5
	(3) Headache – vertigo	44	12.2
	(4) Asthma – bronchitis	42	11.6
	(5) Convulsion – schizophrenia	38	10.5

### Similarity maps of dermatomes, traditional actions, and contemporary indications

**Figure 2:**
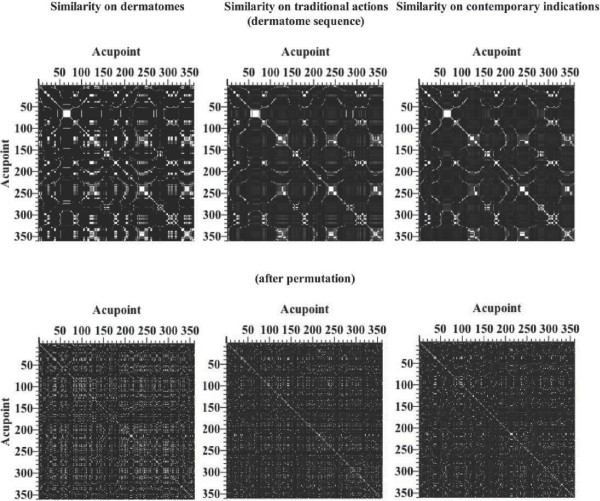
**Similarity maps of channel acupoints calculated based on dermatome information, traditional actions, and contemporary indications.** Top row: Similarity maps of dermatomes (left), traditional actions (middle), and contemporary indications (right) arranged in top-down sequence of dermatomes and channels. Bottom row: Similarity maps of dermatomes (left), traditional actions (middle), and contemporary indications (right) arranged after shuffling. Grey-scale colours indicate variations from low (blackish) to high (whitish) values of similarity.

Similarity maps for matrices *D*, *T*, and *C* (left, middle, right, respectively) in dermatome/channel arrangement and after permutation (top and bottom row, respectively) were exhibited in Figure 2. The white diagonal line evidences the perfect similarity between dual acupoints where *i* = *j*. The similarity maps arranged in dermatome sequence showed several high-similarity small clusters (whitish regions) of dual acupoints spread in a large low-similarity background area (blackish regions). Such clustering was not evident on the similarity maps after permutation of lines and columns, if existent.

### Association between dermatomes and therapeutic characteristics of channel acupoints

Cross-tables from simultaneous classifications of all dual acupoints into levels of similarity were presented in Table 2. Most of dual acupoints presented null to negligible level of similarity in all tested combinations of variables (top-left cell in each cross-table). A decrease in the frequency of acupoints as a function of decreasing levels of similarity calculated from dermatomes versus traditional actions, dermatomes versus contemporary indications, and traditional actions versus contemporary indications. Only a minority of dual acupoint was labelled as very strong similarity.

**Table 2:** Cross-tables of dual acupoints classified by similarity

**Dual acupoint similarity on:**	**Traditional action**						
	**Category**	**Null to negligible**	**Very weak**	**Weak**	**Moderate**	**Strong**	**Very strong**
** Dermatome**	Null to negligible	118872	5763	440	23	12	0
Calculated: γ = 0.542; γ*^2^ ≈ 16%	Very weak	223	14	5	0	0	0
Bootstrap estimation: γ = 0.446 [0.444; 0.447]	Weak	1460	187	27	2	2	0
	Moderate	2166	372	58	3	0	0
Permutation test:γ = 0.002 [0.001; 0.006]	Strong	259	64	8	0	0	0
	Very strong	0	0	0	0	0	0
	**Contemporary indication**						
** Dermatome**	Null to negligible	120799	4083	206	22	0	0
Calculated: γ = 0.657; γ*^2^ ≈ 25%	Very weak	215	27	0	0	0	0
Bootstrap estimation: γ = 0.622 [0.621; 0.623]	Weak	1447	212	17	2	0	0
	Moderate	2197	379	17	2	2	2
Permutation test: γ = −0.001 [−0.006; 0.002]	Strong	254	75	2	0	0	0
	Very strong	0	0	0	0	0	0
	**Contemporary indication**						
** Traditional action**	Null to negligible	119094	3706	172	6	0	2
Calculated: γ = 0.716; γ*^2^ ≈ 30%	Very weak	5444	898	48	8	2	0
Bootstrap estimation: γ = 0.716 [0.715; 0.719]	Weak	360	160	16	2	0	0
	Moderate	12	8	6	2	0	0
Permutation test: γ = 0.000 [−0.001; 0.005]	Strong	2	4	0	8	0	0
	Very strong	0	0	0	0	0	0

As compared to the permutation test, the Goodman-Kruskal γ (Table 2) revealed a significant association between acupoints simultaneously grouped by the strength of similarity of dermatomes and traditional actions (γ = 0.542; *P* < 0.001), as well as by the strength of similarity of dermatomes and contemporary indications (γ = 0.657; *P* < 0.001). A significant association between acupoints simultaneously grouped by the strength of similarities of traditional actions and contemporary indications (γ = 0.716; *P* < 0.001) was also observed. Similarities of dermatomes explained approximately 16% (bootstrap γ*^2^ = 10.5% [10.4; 10.6]) of the variance of traditional actions and 25% (bootstrap γ*^2^ = 21.7% [21.6; 21.8]) of contemporary indications. Traditional actions explained 30% (bootstrap γ*^2^ = 30.2% [30.1; 30.5]) of the variance of contemporary indications.

The bootstrap resamples also showed significant differences (all *P* < 0.001) between all pairs of variables in cross-tables (Table 2). The association between the strength of similarity of traditional actions and contemporary indications was the highest (bootstrap γ = 0.716, CI 95% = [0.715; 0.719]), followed by the association of the strength of similarity of dermatomes and contemporary indications (bootstrap γ = 0.622, 95% CI = [0.621; 0.623]), and finally between the strength of association of dermatomes and traditional actions (bootstrap γ = 0.446, CI 95% = [0.444; 0.447]).

## Discussion

In this study, we investigated the association between the similarity of all channel acupoints with their dermatomes, traditional actions, and contemporary indications. To our knowledge, this is the first study to simultaneously explore the relationship between all channel acupoints’ anatomical and therapeutic characteristics for elucidating the role of the nervous system in the acupuncture stimulation. We found that the dermatomes played a role in the determination of both traditional actions and contemporary indications of channel acupoints, based on the following three main results from this study.

### Similarity of dermatomes partially determines the similarity of therapeutic characteristics of channel acupoints

The results suggested that two acupoints shared dermatomes, traditional actions, and contemporary indications in a directly proportional variation that the explained variation was higher for contemporary indications than for traditional actions. Those associations, besides being moderate-to-strong and accompanied by moderate coefficients of determination, were clinically relevant since it represented the analysis of all acupoints on the channel network. These results were explained by the spatial arrangement of dermatomes and channels on the body surface, and the strength of dual acupoints similarity.

The observed association might be attributed to the differences in the organization of the channel network and dermatomes over the body surface. On the one hand, the channel network is predominantly organized along the vertical axis of the body [5]. On the other hand, the dermatomes system predominantly follows a rostral-caudal distribution of the nervous system with horizontal body segments [34,40]. Hence, there was no theoretical link between the organizational systems of channels and dermatomes to generate expectations on high similarities between dermatomes and traditional actions of dual acupoints. The small number of dual acupoints that actually present some degree of similarity based on these variables could be a consequence of these systematic differences, and supported the partial role of the dermatomes in the determination of traditional actions of acupoints. Nevertheless, the observation that the association between dermatomes and contemporary actions were stronger than between dermatomes and traditional actions required further clarification.

The partial role of dermatomes observed in this study considering all channel acupoints resolved the contradiction of studies regarding a subset of acupoints showing negligible, partial, or strong relationship between dermatomes and therapeutic characteristics of acupoints [7,18-23]. Discrepancies in the observed relationship between dermatomes and therapeutic characteristics of acupoints were due to the applied methods for investigation of the relationship and the coverage of channel acupoints, while most previous studies [18-23] investigated acupoints located in the trunk (*i.e. shu, mu* and *Huatuo-Jiaji* acupoints), where a major spatial relationship between the acupoint skin loci and the visceral efferent innervation was expected.

The relationship between the high-density mesh of acupoints and the therapeutic characteristics of acupoints should be focus of further in-deep investigation. For instance, considered a BSA = 1.79 m^2^ of a subject with height = 1.70 m and weight = 70.0 kg [41]. Considered also the total of acupoints spread over the BSA as 295 (25 midline + 2 × 135 bilateral) in the Yellow Emperor’s Inner Classic and as 670 (52 midline + 2 × 309 bilateral) in the Golden Mirror of Medicine [5]. Therefore, the average skin area of a subject for finding an acupoint was reduced from approximately 61 cm^2^ (7.8 × 7.8 cm) to 27 cm^2^ (5.2 × 5.2 cm) in the referred classic books. This average area would be much lesser, around 12 cm^2^ (3.5 × 3.5 cm), if 386 ‘new’ and ‘off-channel’ bilateral acupoints were also summed [5]. Although these values were not representative for all acupoints because of the actual channel paths along the body surface and the variable inter-acupoint distance along channels, they provided estimations on how close the acupoints were on the body surface – as a matter of fact, some acupoints are indeed located closer than this distance. In combination with a previous study [29] showing that the location of acupoints by CM experts demonstrated variances in range 2.7 cm^2^ to 41.4 cm^2^ for their location, many ‘newly discovered’ acupoints after the Yellow Emperor’s Inner Classic could be mainly due to anatomical variations on the exact location of acupoints lying inside the same dermatome. In view of this, the investigation of the actual distance between dual acupoints over the BSA and its relationship with therapeutic characteristics of acupoints is necessary.

Much effort was directed into research on pattern differentiation for improvement of the efficacy of CM intervention [42], including many automated methods for diagnosing a patient [43]. Based on the results of this study, researchers should also consider the significant relationship between dermatome and therapeutic characteristics of acupoints when planning controlled clinical trials on the efficacy of acupuncture stimulation – and probably other channel-based therapies. The high density-mesh of acupoints on the BSA and the variation on the exact location of acupoints suggested a larger distance between *verum* and sham acupoints for testing their therapeutic effects.

### Association between traditional actions and contemporary indications of acupoints

The level of similarity between dual acupoints based on traditional actions partially determined the level of similarity based on the contemporary indications, being this association the stronger one. The pattern differentiation process in CM was used to guide the treatment of many diseases from all body systems [42,44]. For instance, target-organ damage in patients with systemic arterial hypertension was strongly associated to specific patterns in CM [45] and might aid the selection of the best therapeutic intervention with respect to the Integrative Medicine. Also, it was shown that post-stroke patients exhibited frequency distribution of specific patterns as compared to healthy subjects [46]. Therefore, the either empirical or systematic methods used in the consulted literature [30-33] for derivation of the contemporary indications of acupoints suggested a more consistent relationship between these two therapeutic characteristics than between each one and the dermatomes – a relationship that is dependent of the biologic phenomenon itself. This result calls for a careful revision and urgent update of contemporary CM literature that describes therapeutic intervention for diseases without a clear evidence-based scientific background.

The acupuncture stimulation comprises a nociceptive stimulus from the combination of needle insertion through the dermis and subcutaneous muscles, needle manipulation promoting the mechanical coupling between the needle and connective tissue [47,48], and a significant higher pull-out force for needle extraction [49]. Altogether, these sensory stimuli excite several afferent nerve types [50] as evidenced by the variety of subjective report of the *deqi* sensation [51]. Although it was shown a somatotopic representation of some acupoints in the human primary somatosensory cortex [52], the autonomic responses to a nociceptive stimulus is characterized by patterns of cardiovascular and motor changes [53]. Thus, future experimental studies should investigate if stimulation of acupoints with similar dermatomes may actually evoke proportionally similar physiological responses in internal organs, and concentrate on how it is possible to stimulate the same dermatomes to obtain different therapeutic actions with applications to contemporary indications.

### Analysis of literature compilation content: co-occurrence of terms

“Dispels *wind*” was the most frequent traditional action among all acupoints. *Wind* is the common source for many diseases [54] and is characterized by manifestations with sudden arousal and quick changes, such as muscle spams, vertigo and pain that often changes its location [5]. *Wind* is not only the external cause of a pattern itself, but also may help other climatic factors such as *heat* (*huo*) to penetrate the exterior body [5] – notice that “dispels *heat*” occupies the 2nd position as the top-cited unique term. Likewise, the co-occurrence of “dispels *wind*–dispels *heat*” as the most frequent dual traditional actions was neither surprising. These results reflected either the relevance of the traditional concepts of Six Excess to characterize patterns and the prescription of acupuncture intervention based on pattern differentiation.

Headache was the most frequent contemporary indication, while “asthma–cough” was the most frequent dual term. Three of the top-five terms – headache, low back pain, and vomiting – were amongst the symptoms for which acupuncture was proved through controlled trials to be an effective treatment [4], whereas the 3rd top-ranked term asthma was listed a conditions for which the therapeutic effect of acupuncture has been shown but for which further proof was required. The 5th top-ranked term convulsion was listed under the conditions for which acupuncture may be tried provided the practitioner has special modern medical knowledge and adequate monitoring equipment [4]. These findings reinforced the need for retaining only evidence-based contemporary indications for education of health professionals on CM.

The co-occurrence of “dispels *wind*–headache” might be explained by several factors. Firstly, headache and many related disorders are among the most frequent disorders estimated to occur in 47 % of the adult population at least once a year [52], and any disturbance in *qi* circulation may result in headache [55], but in particular the excess from external *Wind* and *Heat*, among others [5]. Therefore, CM scholars might find acupoints that resulted in the promotion of a healthy flow of *qi* for amelioration of headaches as related to *Wind* and *Heat* as individual or combined factors. The underlying mechanisms of the therapeutic action of headache by acupuncture stimulation were still unclear and required further research.

### Implications for clinical practice

From the clinical point-of-view, the observed role of dermatomes in the determination of therapeutic characteristics of acupoints reinforced the strategy of simultaneously selecting local, distant, and specific acupoints along with painful, *ashi* points [5]. The use of one or more local acupoints over the same dermatomes might evoke the ‘spatial summation’ phenomenon as observed for pain perception [56]. The acupuncture stimulation of one or more distant acupoints located in different dermatomes might cause the ‘whole system’ therapeutic effect in which multiple targets at both visceral and central nervous systems were activated to promote homeostasis. The use of one or more specific acupoints was encouraged that the therapeutic characteristics were effective in high-quality controlled trials.

### Study limitations

The natural language permitting ambiguities within medical terms makes the development of an acupoints dataset challenging. The manual annotation used in this work might introduce errors in the descriptions of therapeutic characteristics of acupoints. Nevertheless, it is believed that all typos were resolved and most of the ambiguities minimized after the revisions for quality control procedure.

All traditional actions and contemporary indications were assumed to reflect the current knowledge and therapeutically effective. This assumption might not be true because many of the contemporary indications have not been tested in double-blinded, controlled randomized clinical trials to be included as an actual contemporary indication to acupuncture [4]. Indeed, no explanations were provided in the consulted atlases concerning the source of the descriptions of therapeutic characteristics, *i.e.* consulted classic books, research studies, clinical experience, among others. Some of the therapeutic characteristics were noise originated from personal experience of the authors, placebo effect, translation errors, transcription errors, and typos. However, this assumption was valid since it simulated how health practitioners used these atlases: they consulted such books to acquire knowledge on acupoints’ anatomic and therapeutic characteristics regardless of the source of this knowledge. Despite the lack of a clear distinction between real and noisy information, the results reflected the current knowledge on the role of the dermatomes for determining the therapeutic characteristics of acupoints. Future studies should test whether or not the selection of a subset of therapeutic characteristics (both traditional and contemporary ones) affected the similarity-based association between therapeutic characteristics of acupoints and their respective dermatomes, in special those subsets that were selected based on high-quality scientific evidence.

The assumption that the dermatomes were correct, constant, and the same in every subject must be discussed as well. Such assumption might not be true because of the variations among atlases of human anatomy, mainly due to known differences in the methods for assessment of their dermatome maps [17,41]. Likewise, there were departures from the anatomical norm of dermatomes on individual subjects and variations throughout the subject’s life spam. Altogether, these criticisms might affect the association. Nevertheless, most health professionals probably disregard these issues during their acupuncture intervention. As the anatomy science was also evolving into an evidence-based approach and new dermatome maps were being generated [40], the impact of dermatome variety should be considered in future studies.

## Conclusions

The similarity of dermatomes between dual acupoints partially determined the similarity of traditional actions and contemporary indications, dermatomes partially determine in the therapeutic efficacy of acupuncture stimulation.

## Abbreviations

AD: *Anno domini*; B: Number of replications in resampling; BC: Before Christ; BSA: Body surface area; C361,361: Similarity matrix of variable contemporary indications; CI 95%: 95% confidence interval; CSV: Comma separated value; D361,361: Similarity matrix of variable dermatomes; Ji, j: Jaccard coefficient of similarity between acupoints *i* and *j*; Ni: Number of terms contained in acupoint *i*; Nij: Number of terms contained in both acupoints; Nj: Number of terms contained in acupoint *j*; T361,361: Similarity matrix of variable traditional actions; γ: Goodman-Kruskal gamma; γ*2: Squared value of the variant of the Goodman-Kruskal gamma.

## Competing interests

The authors declare that they have no competing interests.

## Authors' contributions

ASF and ABL designed and conceived the study. ASF and ABL performed the experiments. ASF and ABL performed the statistical analysis. ASF and ABL wrote the manuscript. Both authors revised and approved the final version of the manuscript.

## Supplementary Material

Additional file 1 Complete dataset of acupoints in Portuguese (Brazil) language. Click here for file

Additional file 2 Computational routines for statistical analysis of acupoints dataset. Click here for file

Additional file 3 Computational routines in editable mode for statistical analysis of acupoints dataset. Click here for file
