# Supplementary material for: Role of dermatomes in the determination of therapeutic characteristics of channel acupoints: a similarity-based analysis of data compiled from literature
Source: Chin Med. 2013 Dec 17;8:24. doi: 10.1186/1749-8546-8-24 (PMC4129182; doi:10.1186/1749-8546-8-24)
Supplement: Additional file 2 — Computational routines for statistical analysis of acupoints dataset. [file 1749-8546-8-24-S2.pdf]

Therapeutic characteristics of channel acupuncture points are associated to dermatomes: A similarity-based analysis from literature compilation

Additional file - Maple worksheet for data analysis
Last update: March 16, 2013.

PS: Most routines may take several hours to complete. The complete worksheet may take several days to complete.

```
> # PART 1A: DESCRIPTIVE ANALYSIS OF ACUPOINTS DATASET:
  DATASET CONTENT (N AND %)
> # Loading packages...
> restart:
> with(ExcelTools):
  with(ListTools):
  with(StringTools):
  with(ArrayTools):
  interface(warnlevel = 0):
> acupoints := 361:
> colunas := Array(["A", "B", "C", "D", "E", "F", "G", "H", "I", "J", "K", "L", "M", "N",
      "O", "P", "Q", "R", "S"]):
> printlevel := 1:
  print("Reading data..."):
  for i from 1 by 1 to ArrayNumElems(colunas) do
    temp1 := "":
    L := "1" : C := colunas[i] : cell := Join([C, L, ":", C, L], ""):
    labels := (Import("Additional file 1.xls", "Acupoints", cell))[1][1]:
    for j from 2 by 1 to acupoints + 1 do
     L := convert(j, string) : C := colunas[i] : cell := Join([C, L, ":", C, L], ""):
     temp2 := (Import("Additional file 1.xls", "Acupoints", cell))[1][1]:
     if temp2 = "-" then temp1 := temp1 : else temp1 := Join([convert(temp1, string), ", ",
       convert(temp2, string)]) : end if:
    end do:
    temp3 := convert(Split(DeleteSpace(temp1), ","), Array):
    temp3 := temp3[2..ArrayNumElems(temp3)]:
    n := ArrayNumElems(convert(MakeUnique(convert(temp3, list)), Array)):
    print(Join([convert(labels, string), ": ", convert(n, string)])):
    output := Vector[row]([labels, n]):
    L := convert(i, string) : cell := Join(["A", L, ":", "B", L], ""):
    Export(output, "Results.xls", "descriptive", cell):
  end do:
  print("End of computation."):
```

```
> # PART 1B: DESCRIPTIVE ANALYSIS OF ACUPOINTS DATASET:
  FREQUENCY AND CO-OCCURRENCE OF UNIQUE AND DUAL TERMS
> # Loading packages...
> restart:
> with(ExcelTools):
  with(ListTools):
  with(StringTools):
  with(ArrayTools):
  interface(warnlevel = 0):
  Digits := 10:
> acupoints := 361:
> # Identification of top-five single terms: Traditional actions
> colunas := Array(["R"]):
  termos5 := Array(1 ..5):
> printlevel := 1:
  for i from 1 by 1 to ArrayNumElems(colunas) do
   print("Reading data and generating a list of unique terms..."):
   temp1 := "":
   for j from 2 by 1 to acupoints + 1 do
    L := convert(j, string) : C := colunas[i] : cell := Join([C, L, ":", C, L], ""):
    temp2 := (Import("Additional file 1.xls", "Acupoints", cell))[1][1]:
    if temp2 = "-" then temp1 := temp1 : else temp1 := Join([convert(temp1, string), ", ",
      convert(temp2, string)]) : end if:
   end do:
   temp3 := MakeUnique(convert(convert(Split(DeleteSpace(temp1), ","), Array), list)):
   temp4 := convert(Split(DeleteSpace(temp1), ","), Array):

   print(Join(["Completed... Starting data analysis of",
     convert(ArrayNumElems(convert(temp3, Array)) - 1, string), "unique terms..."])):
   for k from 1 by 1 to ArrayNumElems(convert(temp3, Array)) do
     n := 0:
     for q from 1 by 1 to ArrayNumElems(convert(temp4, Array)) do
       if temp3[k] = temp4[q] then n := n + 1 else n := n end if:
     end do:
     output := Vector[row]([temp3[k], n]):
     L := convert(k, string) : cell := Join(["A", L, ":", "B", L], ""):
     Export(output, "Results.xls", "list1", cell):
   end do:

   print("Completed... Starting data ranking of top-five terms..."):
   cell := Join(["B1:B", convert(ArrayNumElems(convert(temp3, Array)), string)], ""):
   dados1 := Import("Results.xls", "list1", cell):
   dados2 := Statistics[Sort](Reshape(dados1, ArrayNumElems(convert(temp3,
     Array))), order = descending):
   cell := Join(["A1:A", convert(ArrayNumElems(convert(temp3, Array)), string)], ""):
   dados3 := Reshape(Import("Results.xls", "list1", cell),
     ArrayNumElems(convert(temp3, Array))):
   dados5 := dados2[1 ..5]:
```

```
dados6 := (dados5 / acupoints)·100 :
dados7 := convert(Reshape(dados1, ArrayNumElems(convert(temp3, Array))), list) :
for q from 1 by 1 to 5 do
  indice := (FindMaximalElement(dados7, position))[2] :
  termos5[q] := dados3[indice] :
  dados7 := convert(dados7, Array) :
  dados7[indice] := -1 :
  dados7 := convert(dados7, list) :
 end do:
end do:
dados5; dados6; termos5;
print(Join(["Completed... Total of", convert(k − 2, string), "unique terms ranked."])) :
print("End of computation.") :
```

> # Identification of top-five single terms: Contemporary indications

```
> colunas := Array(["S"]) :
termos5 := Array(1 ..5) :
```

```
> printlevel := 1 :
for i from 1 by 1 to ArrayNumElems(colunas) do
 print("Reading data and generating a list of unique terms...") :
 temp1 := "" :
 for j from 2 by 1 to acupoints + 1 do
  L := convert(j, string) : C := colunas[i] : cell := Join([C, L, ":", C, L], "") :
  temp2 := (Import("Additional file 1.xls", "Acupoints", cell))[1][1] :
  if temp2 = "-" then temp1 := temp1 : else temp1 := Join([convert(temp1, string), ", ",
    convert(temp2, string)]) : end if:
 end do:
 temp3 := MakeUnique(convert(convert(Split(DeleteSpace(temp1), ","), Array), list)) :
 temp4 := convert(Split(DeleteSpace(temp1), ","), Array) :

 print(Join(["Completed... Starting data analysis of",
   convert(ArrayNumElems(convert(temp3, Array)) − 1, string), "unique terms..."])) :
 for k from 1 by 1 to ArrayNumElems(convert(temp3, Array)) do
   n := 0 :
   for q from 1 by 1 to ArrayNumElems(convert(temp4, Array)) do
     if temp3[k] = temp4[q] then n := n + 1 else n := n end if:
   end do:
   output := Vector[row]([temp3[k], n]) :
   L := convert(k, string) : cell := Join(["A", L, ":", "B", L], "") :
   Export(output, "Results.xls", "list2", cell) :
  end do:

  print("Completed... Starting data ranking of top-five terms...") :
  cell := Join(["B1:B", convert(ArrayNumElems(convert(temp3, Array)), string)], "") :
  dados1 := Import("Results.xls", "list2", cell) :
  dados2 := Statistics[Sort](Reshape(dados1, ArrayNumElems(convert(temp3,
    Array))), order = descending) :
  cell := Join(["A1:A", convert(ArrayNumElems(convert(temp3, Array)), string)], "") :
  dados3 := Reshape(Import("Results.xls", "list2", cell),
```

```
  ArrayNumElems(convert(temp3, Array))) :
 dados5 := dados2[1..5] :
 dados6 := dados5/acupoints · 100 :
 dados7 := convert(Reshape(dados1, ArrayNumElems(convert(temp3, Array))), list) :
 for q from 1 by 1 to 5 do
   indice := (FindMaximalElement(dados7, position))[2] :
   termos5[q] := dados3[indice] :
   dados7 := convert(dados7, Array) :
   dados7[indice] := -1 :
   dados7 := convert(dados7, list) :
 end do:
end do:
dados5; dados6; termos5;
print(Join(["Completed... Total of", convert(k − 2, string), "unique terms ranked."])) :
print("End of computation.") :
> # Identification of top-five pairs of terms: Traditional vs.
Traditional
> colunas := Array(["R"]) :
termos5 := Array(1..5) :
> printlevel := 1 :
for i from 1 by 1 to ArrayNumElems(colunas) do
print("Reading data and determining the maximum size of the list of pairs of terms (including
    repetitions)...") :
N := 0 :
 for j from 2 by 1 to acupoints + 1 do
   L := convert(j, string) : C := colunas[i] : cell := Join([C, L, ":", C, L], "") :
   temp22 := (Import("Additional file 1.xls", "Acupoints", cell))[1][1] :
   if temp22 = "-" then temp11 := temp11 : else temp11 := convert(temp22, string) end
  if:
   temp00 := MakeUnique(convert(convert(Split(DeleteSpace(temp11), ","), Array),
  list)) :
  if ArrayNumElems(convert(temp00, Array)) = 0 then N := N :
  elif ArrayNumElems(convert(temp00, Array)) = 1 then N := N + 1 : else N := N
   + (ArrayNumElems(convert(temp00, Array)))! / (((ArrayNumElems(convert(temp00, Array))) − 2)!·2!) : end if:
 end do:

 print(Join(["Completed. Reading data and generating a list of pairs from", convert(N,
   string), "terms (including repetitions)..."])) :
 K := 1 :
 Y := Array(1..N) :
 for j from 2 by 1 to acupoints + 1 do
   L := convert(j, string) : C := colunas[i] : cell := Join([C, L, ":", C, L], "") :
   temp2 := (Import("Additional file 1.xls", "Acupoints", cell))[1][1] :
   if temp2 = "-" then temp1 := temp1 : else temp1 := convert(temp2, string) : end if:
   temp0 := MakeUnique(convert(convert(Split(DeleteSpace(temp1), ","), Array),
    list)) :
   for ii from 1 by 1 to (ArrayNumElems(convert(temp0, Array))) − 1 do
     for jj from ii + 1 by 1 to ArrayNumElems(convert(temp0, Array)) do
```

```
      a := min(temp0_ii, temp0_jj) :
      b := max(temp0_ii, temp0_jj) :
      Y_K := Join([a, "-", b]) :
      K := K + 1 :
    end do:
  end do:
end do:
temp3 := MakeUnique(convert(Y, list)) :
temp4 := Y :

print(Join(["Completed... Starting data analysis of",
  convert(ArrayNumElems(convert(temp3, Array)), string), "unique pairs of terms..."])
  ) :
for k from 1 by 1 to ArrayNumElems(convert(temp3, Array)) do
  n := 0 :
  for q from 1 by 1 to ArrayNumElems(convert(temp4, Array)) do
    if temp3_k = temp4_q then n := n + 1 else n := n end if:
  end do:
  output := Vector_row([temp3_k, n]) :
  L := convert(k, string) : cell := Join(["A", L, ":", "B", L], "") :
  Export(output, "Results.xls", "list3", cell) :
end do:

print("Completed... Starting data ranking of top-five pairs of unique terms...") :
cell := Join(["B1:B", convert(ArrayNumElems(convert(temp3, Array)), string)], "") :
dados1 := Import("Results.xls", "list3", cell) :
dados2 := Statistics[Sort](Reshape(dados1, ArrayNumElems(convert(temp3,
  Array))), order = descending) :
cell := Join(["A1:A", convert(ArrayNumElems(convert(temp3, Array)), string)], "") :
dados3 := Reshape(Import("Results.xls", "list3", cell),
  ArrayNumElems(convert(temp3, Array))) :
dados5 := dados2_1..5 :
dados6 := dados5 / acupoints · 100 :
dados7 := convert(Reshape(dados1, ArrayNumElems(convert(temp3, Array))), list) :
for q from 1 by 1 to 5 do
  indice := (FindMaximalElement(dados7, position))[2] :
  termos5_q := dados3_indice :
  dados7 := convert(dados7, Array) :
  dados7_indice := -1 :
  dados7 := convert(dados7, list) :
end do:
end do:
dados5; dados6; termos5;
print(Join(["Completed... Total of", convert(k − 2, string),
  "unique pairs of terms ranked."])) :
print("End of computation.") :
> # Identification of top-five pairs of terms: Contemporary vs.
  Contemporary
```

```
> colunas := Array(["S"]) :
  termos5 := Array(1..5) :
> printlevel := 1 :
  for i from 1 by 1 to ArrayNumElems(colunas) do
   print("Reading data and determining the maximum size of the list of pairs of terms
      (including repetitions)...") :
   N := 0 :
   for j from 2 by 1 to acupoints + 1 do
      L := convert(j, string) : C := colunas_i : cell := Join([C, L, ":", C, L], "") :
      temp22 := (Import("Additional file 1.xls", "Acupoints", cell))[1][1] :
      if temp22 = "-" then temp11 := temp11 : else temp11 := convert(temp22, string) end
     if:
      temp00 := MakeUnique(convert(convert(Split(DeleteSpace(temp11), ","), Array),
     list)) :
      if ArrayNumElems(convert(temp00, Array)) = 0 then N := N :
     elif ArrayNumElems(convert(temp00, Array)) = 1 then N := N + 1 : else N := N
      + (ArrayNumElems(convert(temp00, Array)))! / (((ArrayNumElems(convert(temp00, Array))) - 2)!·2!) : end if:
    end do:

    print(Join(["Completed. Reading data and generating a list of pairs from", convert(N,
      string), "terms (including repetitions)..."])) :
   K := 1 :
   Y := Array(1..N) :
   for j from 2 by 1 to acupoints + 1 do
     L := convert(j, string) : C := colunas_i : cell := Join([C, L, ":", C, L], "") :
     temp2 := (Import("Additional file 1.xls", "Acupoints", cell))[1][1] :
     if temp2 = "-" then temp1 := temp1 : else temp1 := convert(temp2, string) : end if:
     temp0 := MakeUnique(convert(convert(Split(DeleteSpace(temp1), ","), Array),
      list)) :
     for ii from 1 by 1 to (ArrayNumElems(convert(temp0, Array))) - 1 do
       for jj from ii + 1 by 1 to ArrayNumElems(convert(temp0, Array)) do
         a := min(temp0_ii, temp0_jj) :
         b := max(temp0_ii, temp0_jj) :
         Y_K := Join([a, "-", b]) :
         K := K + 1 :
        end do:
      end do:
    end do:
    temp3 := MakeUnique(convert(Y, list)) :
    temp4 := Y :

    print(Join(["Completed... Starting data analysis of",
      convert(ArrayNumElems(convert(temp3, Array)), string), "unique pairs of terms..."])
      ) :
    for k from 1 by 1 to ArrayNumElems(convert(temp3, Array)) do
      n := 0 :
      for q from 1 by 1 to ArrayNumElems(convert(temp4, Array)) do
        if temp3_k = temp4_q then n := n + 1 else n := n end if:
      end do:
```

```
        output := Vector_row([temp3_k, n]) :
        L := convert(k, string) : cell := Join(["A", L, ":", "B", L], "") :
        Export(output, "Results.xls", "list4", cell) :
      end do:

      print("Completed... Starting data ranking of top-five pairs of unique terms...") :
      cell := Join(["B1:B", convert(ArrayNumElems(convert(temp3, Array)), string)], "") :
      dados1 := Import("Results.xls", "list4", cell) :
      dados2 := Statistics[Sort](Reshape(dados1, ArrayNumElems(convert(temp3,
        Array))), order = descending) :
      cell := Join(["A1:A", convert(ArrayNumElems(convert(temp3, Array)), string)], "") :
      dados3 := Reshape(Import("Results.xls", "list4", cell),
        ArrayNumElems(convert(temp3, Array))) :
      dados5 := dados2_(1 ..5) :
      dados6 := dados5/acupoints · 100 :
      dados7 := convert(Reshape(dados1, ArrayNumElems(convert(temp3, Array))), list) :
     for q from 1 by 1 to 5 do
        indice := (FindMaximalElement(dados7, position))[2] :
        termos5_q := dados3_indice :
        dados7 := convert(dados7, Array) :
        dados7_indice := -1 :
        dados7 := convert(dados7, list) :
      end do:
    end do:
    dados5; dados6; termos5;
    print(Join(["Completed... Total of", convert(k − 2, string),
          "unique pairs of terms ranked."])) :
    print("End of computation.") :
> # Identification of top-five pairs of terms: Traditional vs.
  Contemporary
> colunas := Array(["R", "S"]) :
  termos5 := Array(1 ..5) :
> printlevel := 1 :
      print("Reading data and determining the size of the list of pairs of terms (including
      repetitions)...") :
  N := 0 :
    for j from 2 by 1 to acupoints + 1 do
       L := convert(j, string) : C := colunas_1 : cell := Join([C, L, ":", C, L], "") :
       temp22 := (Import("Additional file 1.xls", "Acupoints", cell))[1][1] :
       if temp22 = "-" then temp111 := temp111 : else temp111 := convert(temp22, string)
      end if:
       temp001 := MakeUnique(convert(convert(Split(DeleteSpace(temp111), ","),
      Array), list)) :
       L := convert(j, string) : C := colunas_2 : cell := Join([C, L, ":", C, L], "") :
       temp22 := (Import("Additional file 1.xls", "Acupoints", cell))[1][1] :
       if temp22 = "-" then temp112 := temp112 : else temp112 := convert(temp22, string)
      end if:
       temp002 := MakeUnique(convert(convert(Split(DeleteSpace(temp112), ","),
      Array), list)) :
```

```
    if ArrayNumElems(convert(temp001, Array))
    = 0 OR ArrayNumElems(convert(temp002, Array)) = 0 then N := N:
   elif ArrayNumElems(convert(temp001, Array))
    = 1 AND ArrayNumElems(convert(temp002, Array)) = 1 then N := N + 1 : else N
    := N + ArrayNumElems(convert(temp001, Array))
   ·ArrayNumElems(convert(temp002, Array)) : end if:
 end do:

print(Join(["Completed. Reading data and generating a list of pairs from", convert(N,
   string), "terms (including repetitions)..."])) :
K := 1 :
Y := Array(1..N) :
for j from 2 by 1 to acupoints + 1 do
  L := convert(j, string) : C := colunas[1] : cell := Join([C, L, ":", C, L], "") :
  temp21 := (Import("Additional file 1.xls", "Acupoints", cell))[1][1] :
  if temp21 = "-" then temp11 := temp11 : else temp11 := convert(temp21, string) : end
   if:
  temp01 := MakeUnique(convert(convert(Split(DeleteSpace(temp11), ","), Array),
   list)) :
  L := convert(j, string) : C := colunas[2] : cell := Join([C, L, ":", C, L], "") :
  temp22 := (Import("Additional file 1.xls", "Acupoints", cell))[1][1] :
  if temp22 = "-" then temp12 := temp12 : else temp12 := convert(temp22, string) : end
   if:
  temp02 := MakeUnique(convert(convert(Split(DeleteSpace(temp12), ","), Array),
   list)) :
  for ii from 1 by 1 to ArrayNumElems(convert(temp01, Array)) do
    for jj from 1 by 1 to ArrayNumElems(convert(temp02, Array)) do
      a := min(temp01[ii], temp02[jj]) :
      b := max(temp01[ii], temp02[jj]) :
      Y[K] := Join([a, "-", b]) :
      K := K + 1 :
    end do:
  end do:
 end do:
 temp3 := MakeUnique(convert(Y, list)) :
 temp4 := Y :

print(Join(["Completed... Starting data analysis of",
   convert(ArrayNumElems(convert(temp3, Array)), string), "pairs of terms..."])) :
 for k from 1 by 1 to ArrayNumElems(convert(temp3, Array)) do
   n := 0 :
   for q from 1 by 1 to ArrayNumElems(convert(temp4, Array)) do
     if temp3[k] = temp4[q] then n := n + 1 else n := n end if:
   end do:
   output := Vector[row]([temp3[k], n]) :
   L := convert(k, string) : cell := Join(["A", L, ":", "B", L], "") :
   Export(output, "Results.xls", "list5", cell) :
 end do:

 print("Completed... Starting data ranking of top-five pairs of unique terms...") :
```

```
cell := Join(["B1:B", convert(ArrayNumElems(convert(temp3, Array)), string)], "") :
dados1 := Import("Results.xls", "list5", cell) :
dados2 := Statistics[Sort](Reshape(dados1, ArrayNumElems(convert(temp3,
  Array))), order = descending) :
cell := Join(["A1:A", convert(ArrayNumElems(convert(temp3, Array)), string)], "") :
dados3 := Reshape(Import("Results.xls", "list5", cell),
  ArrayNumElems(convert(temp3, Array))) :
dados5 := dados2[1..5] :
dados6 := dados5/acupoints·100 :
dados7 := convert(Reshape(dados1, ArrayNumElems(convert(temp3, Array))), list) :
for q from 1 by 1 to 5 do
  indice := (FindMaximalElement(dados7, position))[2] :
  termos5[q] := dados3[indice] :
  dados7 := convert(dados7, Array) :
  dados7[indice] := -1 :
  dados7 := convert(dados7, list) :
end do:
dados5; dados6; termos5;
print(Join(["Completed... Total of", convert(k − 1, string),
    "unique pairs of terms ranked."])) :
print("End of computation.") :
```

```
> # PART 2: DATASET READING AND COMPUTATION OF SIMILARITY
  INDEX AND MATRICES (DATASET SEQUENCE)
> # Loading packages...
> restart :
  st := time_real( ) :
> with(ExcelTools) :
  with(ListTools) :
  with(StringTools) :
  with(plots) :
  with(ArrayTools) :
  interface(warnlevel = 0) :
> # Variables: dermatomes (column P), traditional actions (column
  R), and contemporary indications (column S) -> deterministic
> # Initializing matrices
> acupoints := 361 :
  M1 := Matrix(1 ..acupoints, 1 ..acupoints) :
  M2 := Matrix(1 ..acupoints, 1 ..acupoints) :
  M3 := Matrix(1 ..acupoints, 1 ..acupoints) :
> # Nested "for loop" for calculation of the Jaccard coefficient
  (Ja)
> printlevel := 1 :
  for i from 2 by 1 to acupoints + 1 do
    print(Join(["Running acupoint #", convert(i − 1, string),
      "-----------------------------------------------------------------"])) :

    L := convert(i, string) : C := ("P") : cell := Join([C, L, ":", C, L], "") :
    A1 := (Import("Additional file 1.xls", "Acupoints", cell))[1][1] :
    S1i := ArrayNumElems(convert(Split(DeleteSpace(A1), ","), Array)) :

    L := convert(i, string) : C := ("R") : cell := Join([C, L, ":", C, L], "") :
    B1 := (Import("Additional file 1.xls", "Acupoints", cell))[1][1] :
    S2i := ArrayNumElems(convert(Split(DeleteSpace(B1), ","), Array)) :

    L := convert(i, string) : C := ("S") : cell := Join([C, L, ":", C, L], "") :
    C1 := (Import("Additional file 1.xls", "Acupoints", cell))[1][1] :
    S3i := ArrayNumElems(convert(Split(DeleteSpace(C1), ","), Array)) :

    for j from 2 by 1 to acupoints + 1 do
      L := convert(j, string) : C := ("P") : cell := Join([C, L, ":", C, L], "") :
      A2 := (Import("Additional file 1.xls", "Acupoints", cell))[1][1] :
      S1j := ArrayNumElems(convert(Split(DeleteSpace(A2), ","), Array)) :

      L := convert(j, string) : C := ("R") : cell := Join([C, L, ":", C, L], "") :
      B2 := (Import("Additional file 1.xls", "Acupoints", cell))[1][1] :
      S2j := ArrayNumElems(convert(Split(DeleteSpace(B2), ","), Array)) :

      L := convert(j, string) : C := ("S") : cell := Join([C, L, ":", C, L], "") :
      C2 := (Import("Additional file 1.xls", "Acupoints", cell))[1][1] :
      S3j := ArrayNumElems(convert(Split(DeleteSpace(C2), ","), Array)) :

      E1 := convert(Split(DeleteSpace(Join([A1, ",", A2])), ","), list);
```

```
E2 := convert(Split(DeleteSpace(Join([B1, ",", B2])), ","), list);
E3 := convert(Split(DeleteSpace(Join([C1, ",", C2])), ","), list);

C1ij := ArrayNumElems(convert(FindRepetitions(E1), Array)) :
C2ij := ArrayNumElems(convert(FindRepetitions(E2), Array)) :
C3ij := ArrayNumElems(convert(FindRepetitions(E3), Array)) :

N1 := ArrayNumElems(convert(E1, Array)) :
N2 := ArrayNumElems(convert(E2, Array)) :
N3 := ArrayNumElems(convert(E3, Array)) :
```

$$Ja1 := evalf\left(\frac{C1ij}{S1i + S1j - C1ij}\right) :$$
$$Ja2 := evalf\left(\frac{C2ij}{S2i + S2j - C2ij}\right) :$$
$$Ja3 := evalf\left(\frac{C3ij}{S3i + S3j - C3ij}\right) :$$

```
if i = j then M1[i - 1, j - 1] := 0 : M2[i - 1, j - 1] := 0 : M3[i - 1, j - 1] := 0 :
else M1[i - 1, j - 1] := Ja1 : M2[i - 1, j - 1] := Ja2 : M3[i - 1, j - 1] := Ja3 :
  end if:

end do:
T := convert(time[real]() - st, 'units', 'seconds', 'minutes') :
print(Join(["Processing time: ", convert(T, string), "minutos"])) :

end do:
  print(Join(["-------------------------------------------------------------------Analyses
  completed."]));
> # Storing matrices for offline analyses
> ExportMatrix("M1data.txt", M1, target = delimited, mode = ascii) :
  ExportMatrix("M2data.txt", M2, target = delimited, mode = ascii) :
  ExportMatrix("M3data.txt", M3, target = delimited, mode = ascii) :
```

```
> # PART 3: EXAMPLES
> # EXAMPLE 1
> # Loading packages...
> restart:
> with(ExcelTools):
  with(StringTools):
  interface(warnlevel = 0):
> # Defining indexes
> i := 49:
  j := 50:
> # Reading files with matrices
> M1 := ImportMatrix("M1data.txt", source = delimited, datatype = numeric):
  M2 := ImportMatrix("M2data.txt", source = delimited, datatype = numeric):
  M3 := ImportMatrix("M3data.txt", source = delimited, datatype = numeric):
> # Reading data
> # Selected dual acupoint
> A := Import("Additional file 1.xls", "Acupoints", Join(["C", convert(i + 1, string), ":C",
      convert(i + 1, string)], ""))[1][1]:
  B := Import("Additional file 1.xls", "Acupoints", Join(["E", convert(i + 1, string), ":E",
      convert(i + 1, string)], ""))[1][1]:
  C := Import("Additional file 1.xls", "Acupoints", Join(["D", convert(i + 1, string), ":D",
      convert(i + 1, string)], ""))[1][1]:
  acupoint1 := Join([convert(A, string), convert(B, string), convert(C, string)]);
> A := Import("Additional file 1.xls", "Acupoints", Join(["C", convert(j + 1, string), ":C",
      convert(j + 1, string)], ""))[1][1]:
  B := Import("Additional file 1.xls", "Acupoints", Join(["E", convert(j + 1, string), ":E",
      convert(j + 1, string)], ""))[1][1]:
  C := Import("Additional file 1.xls", "Acupoints", Join(["D", convert(j + 1, string), ":D",
      convert(j + 1, string)], ""))[1][1]:
  acupoint2 := Join([convert(A, string), convert(B, string), convert(C, string)]);
> # Dermatomes
> acupoint1 = Import("Additional file 1.xls", "Acupoints", Join(["P", convert(i + 1, string),
      ":P", convert(i + 1, string)], ""));
  acupoint2 = Import("Additional file 1.xls", "Acupoints", Join(["P", convert(j + 1, string),
      ":P", convert(j + 1, string)], ""));
> # Traditional actions
> acupoint1 = Import("Additional file 1.xls", "Acupoints", Join(["R", convert(i + 1, string),
      ":R", convert(i + 1, string)], ""));
  acupoint2 = Import("Additional file 1.xls", "Acupoints", Join(["R", convert(j + 1,
      string), ":R", convert(j + 1, string)], ""));
> # Contemporary indications
> acupoint1 = Import("Additional file 1.xls", "Acupoints", Join(["S", convert(i + 1, string),
      ":S", convert(i + 1, string)], ""));
  acupoint2 = Import("Additional file 1.xls", "Acupoints", Join(["S", convert(j + 1, string),
      ":S", convert(j + 1, string)], ""));
> # Coefficient J
> M1[i,j];
> M2[i,j];
> M3[i,j];
```

```
> # EXAMPLE 2
> # Defining indexes
> i := 49 :
  j := 58 :
> # Reading data
> # Selected dual acupoint
> A := Import("Additional file 1.xls", "Acupoints", Join(["C", convert(i + 1, string), ":C",
      convert(i + 1, string)], ""))[1][1] :
  B := Import("Additional file 1.xls", "Acupoints", Join(["E", convert(i + 1, string), ":E",
      convert(i + 1, string)], ""))[1][1] :
  C := Import("Additional file 1.xls", "Acupoints", Join(["D", convert(i + 1, string), ":D",
      convert(i + 1, string)], ""))[1][1] :
  acupoint1 := Join([convert(A, string), convert(B, string), convert(C, string)]);
> A := Import("Additional file 1.xls", "Acupoints", Join(["C", convert(j + 1, string), ":C",
      convert(j + 1, string)], ""))[1][1] :
  B := Import("Additional file 1.xls", "Acupoints", Join(["E", convert(j + 1, string), ":E",
      convert(j + 1, string)], ""))[1][1] :
  C := Import("Additional file 1.xls", "Acupoints", Join(["D", convert(j + 1, string), ":D",
      convert(j + 1, string)], ""))[1][1] :
  acupoint2 := Join([convert(A, string), convert(B, string), convert(C, string)]);
> # Dermatomes
> acupoint1 = Import("Additional file 1.xls", "Acupoints", Join(["P", convert(i + 1, string),
      ":P", convert(i + 1, string)], ""));
  acupoint2 = Import("Additional file 1.xls", "Acupoints", Join(["P", convert(j + 1, string),
      ":P", convert(j + 1, string)], ""));
> # Traditional actions
> acupoint1 = Import("Additional file 1.xls", "Acupoints", Join(["R", convert(i + 1, string),
      ":R", convert(i + 1, string)], ""));
  acupoint2 = Import("Additional file 1.xls", "Acupoints", Join(["R", convert(j + 1,
      string), ":R", convert(j + 1, string)], ""));
> # Contemporary indications
> acupoint1 = Import("Additional file 1.xls", "Acupoints", Join(["S", convert(i + 1, string),
      ":S", convert(i + 1, string)], ""));
  acupoint2 = Import("Additional file 1.xls", "Acupoints", Join(["S", convert(j + 1, string),
      ":S", convert(j + 1, string)], ""));
> # Coefficient J
> M1[i,j];
> M2[i,j];
> M3[i,j];
```

```
> # PART 4: READING AND COMPUTATION OF ASSOCIATION TABLES
> # Loading packages...
> restart :
> with(ExcelTools) :
  with(ListTools) :
  with(StringTools) :
  with(plots) :
  with(ArrayTools) :
  with(LinearAlgebra) :
  interface(warnlevel = 0) :
> acupoints := 361 :
> classes := 6 :
> Digits := 3 :
> # Reading files with matrices
> M1 := ImportMatrix("M1data.txt", source = delimited, datatype = numeric) :
  M2 := ImportMatrix("M2data.txt", source = delimited, datatype = numeric) :
  M3 := ImportMatrix("M3data.txt", source = delimited, datatype = numeric) :
> # Filling the main diagonal
> for i from 1 by 1 to acupoints do
   for j from 1 by 1 to acupoints do
     if i = j then M1[i, j] := 1 : M2[i, j] := 1 : M3[i, j] := 1 : end if:
   end do:
   end do:
> # Definition of empty arrays for M12, M13, and M23
> M12 := Matrix(1 ..classes, 1 ..classes) :
  M13 := Matrix(1 ..classes, 1 ..classes) :
  M23 := Matrix(1 ..classes, 1 ..classes) :
> # Nested "for loop" for generation of 2D-Histograms
> # Dermatomes and traditional actions
> x := -1 : y := -1 :
  for i from 1 by 1 to acupoints do
    for j from 1 by 1 to acupoints do
      for k from 1 by 1 to classes do
        if (k - 1)/classes ≤ M1[i,j] < k/classes then x := k : end if:
        if (k - 1)/classes ≤ M2[i,j] < k/classes then y := k : end if:
      end do:
      if i ≠ j then M12[x, y] := M12[x, y] + 1 : end if:
    end do:
   end do:
> # Calculation of concordance and discordance in paired
  observations (diagnonal removed)
> M12;
  concordante := 0 :
  for i from 1 by 1 to classes do
    for j from 1 by 1 to classes do
      a := M12[i, j]; b := M12[i + 1 ..classes, j + 1 .. classes]; c := add(add(b[m, n], m = 1 ..classes
        - i), n = 1 ..classes - j);
```

```
    concordante := concordante + a·c;
  end do:
 end do:
discordante := 0 :
for i from 1 by 1 to classes do
  for j from 1 by 1 to classes do
    a := M12[i, classes - j + 1]; b := M12[i + 1 ..classes, 1 .. classes - j]; c := add(add(b[m, n], m
      = 1 ..classes - i), n = 1 ..classes - j);
    discordante := discordante + a·c;
  end do:
 end do:
 GKgammaM12 := evalf((concordante - discordante)/(concordante + discordante)); RgammaM12
      := evalf((sqrt(concordante) - sqrt(discordante))/sqrt(concordante + discordante)); R2likeM12 := RgammaM12^2;
> # Dermatomes and contemporary indications
> x := -1 : y := -1 :
for i from 1 by 1 to acupoints do
  for j from 1 by 1 to acupoints do
    for k from 1 by 1 to classes do
      if (k - 1)/classes ≤ M1[i, j] < k/classes then x := k : end if:
      if (k - 1)/classes ≤ M3[i, j] < k/classes then y := k : end if:
    end do:
    if i ≠ j then M13[x, y] := M13[x, y] + 1 : end if:
   end do:
 end do:
> # Calculation of concordance and discordance in paired
observations (diagnonal removed)
> M13;
concordante := 0 :
for i from 1 by 1 to classes do
  for j from 1 by 1 to classes do
    a := M13[i, j]; b := M13[i + 1 ..classes, j + 1 .. classes]; c := add(add(b[m, n], m = 1 ..classes
      - i), n = 1 ..classes - j);
    concordante := concordante + a·c;
  end do:
 end do:
discordante := 0 :
for i from 1 by 1 to classes do
  for j from 1 by 1 to classes do
    a := M13[i, classes - j + 1]; b := M13[i + 1 ..classes, 1 .. classes - j]; c := add(add(b[m, n], m
      = 1 ..classes - i), n = 1 ..classes - j);
    discordante := discordante + a·c;
  end do:
 end do:
 GKgammaM13 := evalf((concordante - discordante)/(concordante + discordante)); RgammaM13
```

$:= evalf\left(\frac{\text{sqrt}(concordante) - \text{sqrt}(discordante)}{\text{sqrt}(concordante + discordante)}\right); R2likeM13 := RgammaM13^2;$

> # Traditional actions and contemporary indications

> $x := -1 : y := -1 :$

for i **from** 1 **by** 1 **to** acupoints **do**
 for j **from** 1 **by** 1 **to** acupoints **do**
  for k **from** 1 **by** 1 **to** classes **do**
   if $\frac{k-1}{classes} \le M2_{i,j} < \frac{k}{classes}$ **then** $x := k$: **end if**:
   if $\frac{k-1}{classes} \le M3_{i,j} < \frac{k}{classes}$ **then** $y := k$: **end if**:
  end do:
  if $i \ne j$ **then** $M23_{x,y} := M23_{x,y} + 1$: **end if**:
 end do:
end do:

> # Calculation of concordance and discordance in paired observations (diagonal removed)

> $M23;$

$concordante := 0 :$
for *i* **from** 1 **by** 1 **to** *classes* **do**
 for *j* **from** 1 **by** 1 **to** classes **do**
  $a := M23_{i,j}; b := M23_{i+1..classes, j+1..classes}; c := add(add(b[m,n], m = 1..classes - i), n = 1..classes - j);$
  $concordante := concordante + a \cdot c;$
 end do:
end do:
$discordante := 0 :$
for *i* **from** 1 **by** 1 **to** *classes* **do**
 for *j* **from** 1 **by** 1 **to** classes **do**
  $a := M23_{i, classes - j + 1}; b := M23_{i+1..classes, 1..classes - j}; c := add(add(b[m,n], m = 1..classes - i), n = 1..classes - j);$
  $discordante := discordante + a \cdot c;$
 end do:
end do:

$GKgammaM23 := evalf\left(\frac{concordante - discordante}{concordante + discordante}\right); RgammaM23 := evalf\left(\frac{\text{sqrt}(concordante) - \text{sqrt}(discordante)}{\text{sqrt}(concordante + discordante)}\right); R2likeM23 := RgammaM23^2;$

> # Storing matrices...

> *ExportMatrix*("M12data.txt", *M12*, *target* = *delimited*, *mode* = *ascii*) :
ExportMatrix("M13data.txt", *M13*, *target* = *delimited*, *mode* = *ascii*) :
ExportMatrix("M23data.txt", *M23*, *target* = *delimited*, *mode* = *ascii*) :

> # Saving results...

> $labels := Matrix(1..(classes + 1), 1..(classes + 1)) :$

for *k* **from** 1 **by** 1 **to** *classes* **do**
 $x := \frac{k-1}{classes} : y := \frac{k}{classes} :$
 $labels_{1,k+1} := Join([convert(evalf(x), string), "|-", convert(evalf(y), string)]) :$
 $labels_{k+1,1} := labels_{1,k+1} :$

```
end do:
> variaveis := Vector[row](["Goodman-Kruskal gamma", "Rousson gamma", "R2-like"]) :
results1 := Vector[row]([GKgammaM12, RgammaM12, R2likeM12]) :
results2 := Vector[row]([GKgammaM13, RgammaM13, R2likeM13]) :
results3 := Vector[row]([GKgammaM23, RgammaM23, R2likeM23]) :
> Export(labels, "Results.xls", "GKgammaM12", "A1") :
Export(M12, "Results.xls", "GKgammaM12", "B2") :
Export(variaveis, "Results.xls", "GKgammaM12", "I1") :
Export(results1, "Results.xls", "GKgammaM12", "I2") :
> Export(labels, "Results.xls", "GKgammaM13", "A1") :
Export(M13, "Results.xls", "GKgammaM13", "B2") :
Export(variaveis, "Results.xls", "GKgammaM13", "I1") :
Export(results2, "Results.xls", "GKgammaM13", "I2") :
> Export(labels, "Results.xls", "GKgammaM23", "A1") :
Export(M23, "Results.xls", "GKgammaM23", "B2") :
Export(variaveis, "Results.xls", "GKgammaM23", "I1") :
Export(results3, "Results.xls", "GKgammaM23", "I2") :
```

```
> # PART 5A: READING FOR BOOTSTRAP RESAMPLING AND
  ANALYSIS OF GOODMAN-KRUSKAL GAMMA AND EXPLAINED
  VARIANCE
> # Loading packages...
> restart:
> with(ExcelTools):
  with(ListTools):
  with(StringTools):
  with(plots):
  with(ArrayTools):
  with(LinearAlgebra):
  interface(warnlevel = 0):
  with(DynamicSystems):
> acupoints := 361:
> classes := 6:
> # Reading files with matrices
> Lini := convert(2, string) : Lfim := convert(acupoints + 1, string) : C := ("P") : cell
      := Join([C, Lini, ":", C, Lfim], "") :
  DERM := (Import("Additional file 1.xls", "Acupoints", cell)) :
  M1 := ImportMatrix("M1data.txt", source = delimited, datatype = numeric) :
  M2 := ImportMatrix("M2data.txt", source = delimited, datatype = numeric) :
  M3 := ImportMatrix("M3data.txt", source = delimited, datatype = numeric) :
> # Filling the main diagonal
> for i from 1 by 1 to acupoints do
   for j from 1 by 1 to acupoints do
     if i = j then M1[i, j] := 1 : M2[i, j] := 1 : M3[i, j] := 1 : end if:
   end do:
   end do:
> # Analysis by dermatome level
> # Generating unique dermatome levels combinations
> LEVEL := convert(sort(MakeUnique(convert(DERM, list))), Array) :
  N := ArrayNumElems(LEVEL) :
> # "For loop" for sorting acupoints by first dermatome level
> rank := Array(1 ..acupoints) :
  freq := Array(1 ..N) :
> for i from 1 by 1 to acupoints do
    for j from 1 by 1 to N do
     if evalb(DERM[i][1] = LEVEL_j) = true then freq_j := freq_j + 1; rank_i := j else freq_j
       = freq_j; rank_i := rank_i : end if:
    end do:
  end do:
> rankM1 := Matrix(1 ..acupoints, 1 ..acupoints) :
  rankM2 := Matrix(1 ..acupoints, 1 ..acupoints) :
  rankM3 := Matrix(1 ..acupoints, 1 ..acupoints) :
> rank1 := rank:
  for i from 1 by 1 to acupoints do
   for j from 1 by 1 to acupoints do
    rankM1_{i,j} := M1[(max(rank1[i], rank1[j])), (min(rank1[i], rank1[j]))];
    rankM2_{i,j} := M2[(max(rank1[i], rank1[j])), (min(rank1[i], rank1[j]))];
```

```
rankM3[i,j] := M3[(max(rank1[i], rank1[j])), (min(rank1[i], rank1[j]))];
 end do:
end do:
> # Initializing the bootstrap procedure on the ranked matrix
  (previously sorted by dermatome)
> # Real sample size
> N := acupoints :
> # Repeat this sorting B times (idealy: B = 1000 or more)
> B := 1000 :
> # Storing column headers in Excel;
> header := Vector[row](["Goodman-Kruskal gamma", "Rousson gamma", "R2-like"]) :
  Export(header, "Results.xls", "M12", "A1") :
  Export(header, "Results.xls", "M13", "A1") :
  Export(header, "Results.xls", "M23", "A1") :
> # Definition of empty arrays for bivariate analysis
> M12 := Matrix(1 ..classes, 1 ..classes) :
  M13 := Matrix(1 ..classes, 1 ..classes) :
  M23 := Matrix(1 ..classes, 1 ..classes) :
> # M12: Dermatomes vs. Traditional Indications
> # Nested loops for bootstrap calculations (# Dermatomes and
  traditional actions: sampling N dual acupoints with replacement
  B times)
> printlevel := 1 : st := time[real]( ) :
> for q from 1 by 1 to B do
   x := -1 : y := -1 : Seed := randomize( ) :
   for n from 1 by 1 to N^2 do
    L1 := RandomTools[Generate](integer(range = 1 ..acupoints)); L2
      := RandomTools[Generate](integer(range = 1 ..acupoints));
    for k from 1 by 1 to classes do
      if (k - 1)/classes <= M1[L1, L2] < k/classes then x := k : end if:
      if (k - 1)/classes <= M2[L1, L2] < k/classes then y := k : end if:
     end do:
     if L1 <> L2 then M12[x, y] := M12[x, y] + 1 : end if:
   end do:
   concordante := 0 :
   for i from 1 by 1 to classes do
    for j from 1 by 1 to classes do
     a := M12[i, j]; b := M12[i + 1 ..classes, j + 1 .. classes]; c := add(add(b[m, n], m = 1
     ..classes - i), n = 1 ..classes - j);
     concordante := concordante + a·c;
    end do:
   end do:
   discordante := 0 :
   for i from 1 by 1 to classes do
    for j from 1 by 1 to classes do
     a := M12[i, classes - j + 1] : b := M12[i + 1 ..classes, 1 .. classes - j]; c := add(add(b[m, n],
     m = 1 ..classes - i), n = 1 ..classes - j);
```

```
      discordante := discordante + a·c;
    end do:
  end do:
  GKgammaM12 := evalf((concordante − discordante)/(concordante + discordante)) : RgammaM12
     := evalf((sqrt(concordante) − sqrt(discordante))/sqrt(concordante + discordante)) : R2likeM12 := RgammaM12^2 :
  results1 := Vector_row([GKgammaM12, RgammaM12, R2likeM12]) :
  Export(results1, "Results.xls", "M12", StringTools[DeleteSpace](Join(["A", convert(q
     + 1, string)]))) :
  T := convert(time_real() - st, 'units', 'seconds', 'minutes') :
  print(Join([convert(q, string), ") Duração do processamento: ", convert(T, string),
     "minutos"]));
end do:
```

> # M13: Dermatomes vs. Contemporary Indications

> # Nested loops for bootstrap calculations (# Dermatomes and contemporary actions: sampling N dual acupoints with replacement B times)

```
> printlevel := 1 : st := time_real() :
> for q from 1 by 1 to B do
  x := -1 : y := -1 : Seed := randomize() :
  for n from 1 by 1 to N^2 do
    L1 := RandomTools[Generate](integer(range = 1 ..acupoints)); L2
      := RandomTools[Generate](integer(range = 1 ..acupoints));
    for k from 1 by 1 to classes do
      if (k − 1)/classes ≤ M1[L1, L2] < k/classes then x := k : end if:
      if (k − 1)/classes ≤ M3[L1, L2] < k/classes then y := k : end if:
    end do:
    if L1 ≠ L2 then M13[x, y] := M13[x, y] + 1 : end if:
  end do:
  concordante := 0 :
  for i from 1 by 1 to classes do
    for j from 1 by 1 to classes do
      a := M13[i, j]; b := M13[i + 1 ..classes, j + 1 .. classes]; c := add(add(b[m, n], m = 1
      ..classes − i), n = 1 ..classes − j);
      concordante := concordante + a·c;
    end do:
  end do:
  discordante := 0 :
  for i from 1 by 1 to classes do
    for j from 1 by 1 to classes do
      a := M13[i, classes − j + 1] : b := M13[i + 1 ..classes, 1 .. classes − j]; c := add(add(b[m, n],
      m = 1 ..classes − i), n = 1 ..classes − j);
      discordante := discordante + a·c;
    end do:
  end do:
```

```
GKgammaM13 := evalf((concordante - discordante)/(concordante + discordante)) : RgammaM13
    := evalf((sqrt(concordante) - sqrt(discordante))/sqrt(concordante + discordante)) : R2likeM13 := RgammaM13^2 :
results2 := Vector[row]([GKgammaM13, RgammaM13, R2likeM13]) :
Export(results2, "Results.xls", "M13", StringTools[DeleteSpace](Join(["A", convert(q
    + 1, string)]))) :
T := convert(time[real]() - st, 'units', 'seconds', 'minutes') :
print(Join([convert(q, string), ") Duração do processamento: ", convert(T, string),
    "minutos"]));
end do:
```

```
> # M23: Traditional Indications vs. Contemporary
  Indications
> # Nested loops for bootstrap calculations (# traditional and
  contemporary actions: sampling N dual acupoints with
  replacement B times)
> printlevel := 1 : st := time[real]() :
> for q from 1 by 1 to B do
   x := -1 : y := -1 : Seed := randomize() :
   for n from 1 by 1 to N^2 do
    L1 := RandomTools[Generate](integer(range = 1 ..acupoints])); L2
      := RandomTools[Generate](integer(range = 1 ..acupoints));
    for k from 1 by 1 to classes do
      if (k - 1)/classes <= M2[L1, L2] < k/classes then x := k : end if:
      if (k - 1)/classes <= M3[L1, L2] < k/classes then y := k : end if:
     end do:
     if L1 <> L2 then M23[x, y] := M23[x, y] + 1 : end if:
   end do:
   concordante := 0 :
   for i from 1 by 1 to classes do
    for j from 1 by 1 to classes do
     a := M23[i, j]; b := M23[i + 1 ..classes, j + 1 .. classes]; c := add(add(b[m, n], m = 1
     ..classes - i), n = 1 ..classes - j);
     concordante := concordante + a·c;
    end do:
   end do:
   discordante := 0 :
   for i from 1 by 1 to classes do
    for j from 1 by 1 to classes do
     a := M23[i, classes - j + 1] : b := M23[i + 1 ..classes, 1 .. classes - j]; c := add(add(b[m, n],
     m = 1 ..classes - i), n = 1 ..classes - j);
     discordante := discordante + a·c;
    end do:
   end do:
   GKgammaM23 := evalf((concordante - discordante)/(concordante + discordante)) : RgammaM23
```

```
:= evalf( sqrt(concordante) - sqrt(discordante) / sqrt(concordante + discordante) ) : R2likeM23 := RgammaM23^2 :
results3 := Vector_row([GKgammaM23, RgammaM23, R2likeM23]) :
Export(results3, "Results.xls", "M23", StringTools[DeleteSpace](Join(["A", convert(q
    + 1, string)]))) :
T := convert(time_real() - st, 'units', 'seconds', 'minutes') :
print(Join([convert(q, string), ") Duração do processamento: ", convert(T, string),
    "minutos"]));
end do:
```

```
> # PART 5B: DATA ANALYSES OF BOOTSTRAP
  SAMPLES (CALCULATION OF CONFIDENCE INTERVAL)
> restart:
  st := time[real]():
  decimais := 3:
> # Read data from Excel to estimare R2 and CI95%
> with(ExcelTools):
  with(StringTools):
  with(LinearAlgebra):
  with(DynamicSystems):
  with(ArrayTools):
  with(plots):
> # Quantity of matrices to test
> MODEL := 3:
> # Repeat this sorting B times (idealy: B = 1000 or more)
> B := 1000:
> # Definition of the confidence interval
> α := 0.05:
> # Defining variables
> θ := Matrix(1..B, 1..1):
  colunas := ("A", "B", "C"):
  Labels1 := Matrix(["M12", "M13", "M23"]):
  Labels2 := Matrix(["Dermatomes vs. Traditional indications",
      "Dermatomes vs. Contemporary indications",
      "Traditional vs. Contemporary indications"]):
  Labels1 := Labels1[1]:
  Labels2 := Labels2[1]:
  header := Vector[row](["Goodman-Kruskal gamma", "Rousson gamma", "R2-like"]):
  HISTO := Matrix(1..MODEL, 1..3):
> # Defining X-axis limits of histograms
> minimo := Array(1..MODEL):
  maximo := Array(1..MODEL):
> for i from 1 by 1 to MODEL do
    L := convert(2, string) : C := convert(colunas[i], string) : cell := Join([C, L, ":", C,
      convert(B + 1, string)], "") :
    temp1 := Import("Results.xls", "M12", cell) :
    L := convert(2, string) : C := convert(colunas[i], string) : cell := Join([C, L, ":", C,
      convert(B + 1, string)], "") :
    temp2 := Import("Results.xls", "M13", cell) :
    L := convert(2, string) : C := convert(colunas[i], string) : cell := Join([C, L, ":", C,
      convert(B + 1, string)], "") :
    temp3 := Import("Results.xls", "M23", cell) :
    minimo[i] := min(Concatenate(1, temp1, temp2, temp3)) : maximo[i]
       := max(Concatenate(1, temp1, temp2, temp3)) :
  end do:
> # Reading data for generation of histograms and confidence
  intervals
```

```
> resultado := Matrix(1..MODEL·3, 1..5) :
> infolevel[Statistics] := 1 :
  k := 1 :
  for i from 1 by 1 to MODEL do
   for j from 1 by 1 to 3 do
    cells := DeleteSpace(Join([convert(colunas_i, string), "2:", colunas_i, convert(B + 1,
      string)])) :
    θ := sort(convert((Import("Results.xls", Labels1_j, cells))_{1..B, 1}, Vector[column])) :
    Statistics[ShapiroWilkWTest](θ, level = α) :
    mediana := convert(Statistics[Median](θ), float, decimais) :
    low := convert(θ_{floor(B·(α/2))}, float, decimais) :
    up := convert(θ_{ceil(B·(1 - α/2))}, float, decimais) :
    results := StringTools[Join]([convert(Labels1_j, string), ":", convert(header_i, string),
      "=", convert(mediana, string), ", CI95%=[", convert(low, string), ",", convert(up,
      string), "]"]) :
    resultado_{k, 1} := Labels1_j : resultado_{k, 2} := header_i : resultado_{k, 3} := mediana :
      resultado_{k, 4} := low : resultado_{k, 5} := up :
    HISTO_{j, i} := Statistics[Histogram](θ, title = Labels2_j, labels = [header_i,"Frequency (%)
      "], axesfont = [ARIAL, bold, 12], labelfont = [ARIAL, bold, 14], titlefont = [ARIAL,
      bold, 16], labeldirections = ["horizontal", "vertical"], color = gray, range = minimo_i
      ..maximo_i) :
    print(results);
   display(HISTO_{j, 1});
   k := k + 1 :
   end do:
  end do:
> Export(Vector_row(["Model", "Parameter", "Median", "Low 95%CI", "High 95%CI"]),
      "Results.xls", "bootstrap", "A1") :
  Export(resultado, "Results.xls", "bootstrap", "A2") :
> # Plots - Arrays of histograms
> plotsetup(bmp, plotoutput = `Figure1.bmp`, plotoptions
      = `landscape, noborder, height=1500, width=1500`) :
  display(HISTO);
  plotsetup(default) :
  display(HISTO);
```

```
> # PART 6A: READING FOR BOOTSTRAP RESAMPLING AND
  ANALYSIS OF CONFIDENCE INTERVAL FOR GOODMAN-KRUSKAL
  GAMMA AND EXPLAINED VARIANCE (PERMUTATION TEST)
> # Loading packages...
> restart:
> with(ExcelTools):
  with(ListTools):
  with(StringTools):
  with(plots):
  with(ArrayTools):
  with(LinearAlgebra):
  interface(warnlevel = 0):
  with(DynamicSystems):
> acupoints := 361:
> classes := 6:
> # Reading files with matrices
> Lini := convert(2, string) : Lfim := convert(acupoints + 1, string) : C := ("P") : cell
      := Join([C, Lini, ":", C, Lfim], ""):
  DERM := (Import("Additional file 1.xls", "Acupoints", cell)):
  M1 := ImportMatrix("M1data.txt", source = delimited, datatype = numeric):
  M2 := ImportMatrix("M2data.txt", source = delimited, datatype = numeric):
  M3 := ImportMatrix("M3data.txt", source = delimited, datatype = numeric):
> # Filling the main diagonal
> for i from 1 by 1 to acupoints do
   for j from 1 by 1 to acupoints do
    if i = j then M1[i, j] := 1 : M2[i, j] := 1 : M3[i, j] := 1 : end if:
   end do:
   end do:
> # Analysis by dermatome level
> # Generating unique dermatome levels combinations
> LEVEL := convert(sort(MakeUnique(convert(DERM, list))), Array):
  N := ArrayNumElems(LEVEL):
> # "For loop" for sorting acupoints by first dermatome level
> rank := Array(1..acupoints):
  freq := Array(1..N):
> for i from 1 by 1 to acupoints do
    for j from 1 by 1 to N do
     if evalb(DERM[i][1] = LEVEL_j) = true then freq_j := freq_j + 1; rank_i := j else freq_j
       = freq_j; rank_i := rank_i : end if:
    end do:
  end do:
> rankM1 := Matrix(1..acupoints, 1..acupoints):
  rankM2 := Matrix(1..acupoints, 1..acupoints):
  rankM3 := Matrix(1..acupoints, 1..acupoints):
> rank1 := rank:
  for i from 1 by 1 to acupoints do
   for j from 1 by 1 to acupoints do
    rankM1_{i,j} := M1[(max(rank1[i], rank1[j])), (min(rank1[i], rank1[j]))];
    rankM2_{i,j} := M2[(max(rank1[i], rank1[j])), (min(rank1[i], rank1[j]))];
```

```
    rankM3[i, j] := M3[(max(rank1[i], rank1[j])), (min(rank1[i], rank1[j]))];
   end do:
  end do:
> # Initializing the bootstrap procedure on the ranked matrix
  (previously sorted by dermatome)
> # Real sample size
> N := acupoints:
> # Repeat this sorting B times (idealy: B = 1000 or more)
> B := 1000 :
> # Storing column headers in Excel;
> header := Vector[row](["Goodman-Kruskal gamma", "Rousson gamma", "R2-like"]) :
  Export(header, "Results.xls", "M12perm", "A1") :
  Export(header, "Results.xls", "M13perm", "A1") :
  Export(header, "Results.xls", "M23perm", "A1") :
> # Definition of empty arrays for bivariate analysis
> M12 := Matrix(1 ..classes, 1 ..classes) :
  M13 := Matrix(1 ..classes, 1 ..classes) :
  M23 := Matrix(1 ..classes, 1 ..classes) :
> # M12: Dermatomes vs. Traditional Indications
> # Nested loops for bootstrap calculations (# Dermatomes and
  traditional actions: sampling N dual acupoints with replacement
  B times)
> printlevel := 1 : st := time[real]( ) :
> for q from 1 by 1 to B do
   x := -1 : y := -1 : Seed := randomize( ) :
   for n from 1 by 1 to N^2 do
    L1 := RandomTools[Generate](integer(range = 1 ..acupoints)); L2
     := RandomTools[Generate](integer(range = 1 ..acupoints)); L3
     := RandomTools[Generate](integer(range = 1 ..acupoints)); L4
     := RandomTools[Generate](integer(range = 1 ..acupoints));
    for k from 1 by 1 to classes do
     if (k - 1)/classes <= M1[L1, L2] < k/classes then x := k : end if:
     if (k - 1)/classes <= M2[L3, L4] < k/classes then y := k : end if:
    end do:
    if (L1 ≠ L2 AND L3 ≠ L4) then M12[x, y] := M12[x, y] + 1 : end if:
   end do:
   concordante := 0 :
   for i from 1 by 1 to classes do
    for j from 1 by 1 to classes do
     a := M12[i, j]; b := M12[i + 1 ..classes, j + 1 .. classes]; c := add(add(b[m, n], m = 1
     ..classes - i), n = 1 ..classes - j);
     concordante := concordante + a·c;
    end do:
   end do:
   discordante := 0 :
   for i from 1 by 1 to classes do
    for j from 1 by 1 to classes do
```

```
a := M12[i, classes - j + 1] : b := M12[i + 1 ..classes, 1 .. classes - j]; c := add(add(b[m, n],
m = 1 ..classes - i), n = 1 ..classes - j);
discordante := discordante + a·c;
end do:
end do:
GKgammaM12 := evalf((concordante - discordante)/(concordante + discordante)) : RgammaM12
:= evalf((sqrt(concordante) - sqrt(discordante))/(sqrt(concordante + discordante))) : R2likeM12 := RgammaM12^2 :
results1 := Vector[row]([GKgammaM12, RgammaM12, R2likeM12]) :
Export(results1, "Results.xls", "M12perm", StringTools[DeleteSpace](Join(["A",
convert(q + 1, string)]))) :
T := convert(time[real]( ) - st, 'units', 'seconds', 'minutes') :
print(Join([convert(q, string), ") Duração do processamento: ", convert(T, string),
"minutos"]));
end do:
```

```
> # M13: Dermatomes vs. Contemporary Indications
> # Nested loops for bootstrap calculations (# Dermatomes and contemporary actions: sampling N dual acupoints with replacement B times)
> printlevel := 1 : st := time[real]( ) :
> for q from 1 by 1 to B do
x := -1 : y := -1 : Seed := randomize( ) :
for n from 1 by 1 to N^2 do
L1 := RandomTools[Generate](integer(range = 1 ..acupoints)); L2
:= RandomTools[Generate](integer(range = 1 ..acupoints)); L3
:= RandomTools[Generate](integer(range = 1 ..acupoints)); L4
:= RandomTools[Generate](integer(range = 1 ..acupoints));
for k from 1 by 1 to classes do
if (k - 1)/classes ≤ M1[L1, L2] < k/classes then x := k : end if:
if (k - 1)/classes ≤ M3[L3, L4] < k/classes then y := k : end if:
end do:
if L1 ≠ L2 then M13[x, y] := M13[x, y] + 1 : end if:
end do:
concordante := 0 :
for i from 1 by 1 to classes do
for j from 1 by 1 to classes do
a := M13[i, j]; b := M13[i + 1 ..classes, j + 1 .. classes]; c := add(add(b[m, n], m = 1
..classes - i), n = 1 ..classes - j);
concordante := concordante + a·c;
end do:
end do:
discordante := 0 :
for i from 1 by 1 to classes do
for j from 1 by 1 to classes do
a := M13[i, classes - j + 1] : b := M13[i + 1 ..classes, 1 .. classes - j]; c := add(add(b[m, n],
m = 1 ..classes - i), n = 1 ..classes - j);
```

```
      discordante := discordante + a·c;
    end do:
  end do:
  GKgammaM13 := evalf((concordante − discordante)/(concordante + discordante)) : RgammaM13
    := evalf((sqrt(concordante) − sqrt(discordante))/(sqrt(concordante + discordante))) : R2likeM13 := RgammaM13^2 :
  results2 := Vector_row([GKgammaM13, RgammaM13, R2likeM13]) :
  Export(results2, "Results.xls", "M13perm", StringTools[DeleteSpace](Join(["A",
    convert(q + 1, string)]))) :
  T := convert(time_real( ) - st, 'units', 'seconds', 'minutes') :
  print(Join([convert(q, string), ") Duração do processamento: ", convert(T, string),
    "minutos"]));
end do:
```

M23: Traditional Indications vs. Contemporary Indications

Nested loops for bootstrap calculations (# traditional and contemporary actions: sampling N dual acupoints with replacement B times)

```
> printlevel := 1 : st := time_real( ) :
> for q from 1 by 1 to B do
  x := -1 : y := -1 : Seed := randomize( ) :
  for n from 1 by 1 to N^2 do
    L1 := RandomTools[Generate](integer(range = 1 ..acupoints)); L2
      := RandomTools[Generate](integer(range = 1 ..acupoints)); L3
      := RandomTools[Generate](integer(range = 1 ..acupoints)); L4
      := RandomTools[Generate](integer(range = 1 ..acupoints));
    for k from 1 by 1 to classes do
      if (k − 1)/classes ≤ M2[L1, L2] < k/classes then x := k : end if:
      if (k − 1)/classes ≤ M3[L3, L4] < k/classes then y := k : end if:
    end do:
    if (L1 ≠ L2 AND L3 ≠ L4) then M23[x, y] := M23[x, y] + 1 : end if:
  end do:
  concordante := 0 :
  for i from 1 by 1 to classes do
    for j from 1 by 1 to classes do
      a := M23[i, j]; b := M23[i + 1 ..classes, j + 1 .. classes]; c := add(add(b[m, n], m = 1
      ..classes − i), n = 1 ..classes − j);
      concordante := concordante + a·c;
    end do:
  end do:
  discordante := 0 :
  for i from 1 by 1 to classes do
    for j from 1 by 1 to classes do
      a := M23[i, classes − j + 1] : b := M23[i + 1 ..classes, 1 .. classes − j]; c := add(add(b[m, n],
      m = 1 ..classes − i), n = 1 ..classes − j);
      discordante := discordante + a·c;
```

```
        end do:
      end do:
      GKgammaM23 := evalf((concordante - discordante)/(concordante + discordante)) : RgammaM23
        := evalf((sqrt(concordante) - sqrt(discordante))/(sqrt(concordante + discordante))) : R2likeM23 := RgammaM23^2 :
      results3 := Vector[row]([GKgammaM23, RgammaM23, R2likeM23]) :
      Export(results3, "Results.xls", "M23perm", StringTools[DeleteSpace](Join(["A",
        convert(q + 1, string)]))) :
      T := convert(time[real]() - st, 'units', 'seconds', 'minutes') :
      print(Join([convert(q, string), ") Duração do processamento: ", convert(T, string),
        "minutos"]));
    end do:
```

```
> # PART 6B: DATA ANALYSES OF BOOTSTRAP SAMPLES (CONFIDENCE INTERVAL OF PERMUTATION TEST)
> restart:
  st := time[real]( ):
  decimais := 3:
> # Read data from Excel to estimare R2 and CI95%
> with(ExcelTools):
  with(StringTools):
  with(LinearAlgebra):
  with(DynamicSystems):
  with(ArrayTools):
  with(plots):
> # Quantity of matrices to test
> MODEL := 3:
> # Repeat this sorting B times (idealy: B = 1000 or more)
> B := 1000:
> # Definition of the confidence interval
> α := 0.05:
> # Defining variables
> θ := Matrix(1..B, 1..1):
  colunas := ("A", "B", "C"):
  Labels1 := Matrix(["M12perm", "M13perm", "M23perm"]):
  Labels2 := Matrix(["Dermatomes vs. Traditional indications",
      "Dermatomes vs. Contemporary indications",
      "Traditional vs. Contemporary indications"]):
  Labels1 := Labels1[1]:
  Labels2 := Labels2[1]:
  header := Vector[row](["Goodman-Kruskal gamma", "Rousson gamma", "R2-like"]):
  HISTO := Matrix(1..MODEL, 1..3):
> # Defining axes limits of histograms
> minimo := Array(1..MODEL):
  maximo := Array(1..MODEL):
> for i from 1 by 1 to MODEL do
    L := convert(2, string) : C := convert(colunas[i], string) : cell := Join([C, L, ":", C,
      convert(B + 1, string)], "") :
    temp1 := Import("Results.xls", "M12perm", cell) :
    L := convert(2, string) : C := convert(colunas[i], string) : cell := Join([C, L, ":", C,
      convert(B + 1, string)], "") :
    temp2 := Import("Results.xls", "M13perm", cell) :
    L := convert(2, string) : C := convert(colunas[i], string) : cell := Join([C, L, ":", C,
      convert(B + 1, string)], "") :
    temp3 := Import("Results.xls", "M23perm", cell) :
    minimo[i] := min(Concatenate(1, temp1, temp2, temp3)) : maximo[i]
      := max(Concatenate(1, temp1, temp2, temp3)) :
  end do:
> # Reading data for generation of histograms
```

```
> resultado := Matrix(1..MODEL·3, 1..5) :
> infolevel[Statistics] := 1 :
  k := 1 :
  for i from 1 by 1 to MODEL do
   for j from 1 by 1 to 3 do
    cells := DeleteSpace(Join([convert(colunas_i, string), "2:", colunas_i, convert(B + 1,
      string)])) :
    θ := sort(convert((Import("Results.xls", Labels1_j, cells))_{1..B, 1}, Vector[column])) :
    Statistics[ShapiroWilkWTest](θ, level = α) :
    mediana := convert(Statistics[Median](θ), float, decimais) :
    low := convert(θ_{floor(B·(α/2))}, float, decimais) :
    up := convert(θ_{ceil(B·(1 - α/2))}, float, decimais) :
    results := StringTools[Join]([convert(Labels1_j, string), ":", convert(header_i, string),
      "=", convert(mediana, string), ", CI95%=[", convert(low, string), ",", convert(up,
      string), "]"]) :
    resultado_{k, 1} := Labels1_j : resultado_{k, 2} := header_i : resultado_{k, 3} := mediana :
      resultado_{k, 4} := low : resultado_{k, 5} := up :
    HISTO_{j, i} := Statistics[Histogram](θ, title = Labels2_j, labels = [header_i,"Frequency (%)
      "], axesfont = [ARIAL, bold, 12], labelfont = [ARIAL, bold, 14], titlefont = [ARIAL,
      bold, 16], labeldirections = ["horizontal", "vertical"], color = gray, range = minimo_i
      ..maximo_i) :
    print(results);
   display(HISTO_{j, i});
   k := k + 1 :
   end do:
  end do:
> Export(Vector_row(["Model", "Parameter", "Median", "Low 95%CI", "High 95%CI"]),
      "Results.xls", "permutation", "A1") :
  Export(resultado, "Results.xls", "permutation", "A2") :
> # Plots - Arrays of histograms
> plotsetup(bmp, plotoutput = `Figure2.bmp`, plotoptions
      = `landscape, noborder, height=1500, width=1500`) :
  display(HISTO);
  plotsetup(default) :
  display(HISTO);
```

```
> # PART 7: READING AND COMPUTATION OF P-VALUES BETWEEN
  BOOTSTRAP AND PERMUTATION TESTS
> # Loading packages...
> restart :
> with(ExcelTools) :
  with(ListTools) :
  with(StringTools) :
  with(plots) :
  with(ArrayTools) :
  with(LinearAlgebra) :
  interface(warnlevel = 0) :
> # Quantity of matrices to test
> MODEL := 3 :
> # Repeat this sorting B times (idealy: B = 1000 or more)
> B := 1000 :
> # Reading data with calculated G-K-gamma
> M12γ_calculated := Import("Results.xls", "GKgammaM12", "I2:I2")[1][1] :
  M13γ_calculated := Import("Results.xls", "GKgammaM13", "I2:I2")[1][1] :
  M23γ_calculated := Import("Results.xls", "GKgammaM23", "I2:I2")[1][1] :
> # Reading data with bootstrap resampling
> M12γ_bootstrap := Import("Results.xls", "bootstrap", "C2:C2")[1][1] :
  M13γ_bootstrap := Import("Results.xls", "bootstrap", "C3:C3")[1][1] :
  M23γ_bootstrap := Import("Results.xls", "bootstrap", "C4:C4")[1][1] :
> # Reading data with permutated resampling
> M12γ_permutation := Import("Results.xls", "permutation", "C2:C2")[1][1] :
  M13γ_permutation := Import("Results.xls", "permutation", "C3:C3")[1][1] :
  M23γ_permutation := Import("Results.xls", "permutation", "C4:C4")[1][1] :
> # Reading files and calculation of P-values (calculated versus
  permutation)
> n1 := 0 : n2 := 0 : n3 := 0 :
  for i from 1 by 1 to B do
     data12 := (Import("Results.xls", "M12perm", Join(["A", convert(i + 1, string), ":",
      "A", convert(i + 1, string)], ""))[1][1]) :
     data13 := (Import("Results.xls", "M13perm", Join(["A", convert(i + 1, string), ":",
      "A", convert(i + 1, string)], ""))[1][1]) :
     data23 := (Import("Results.xls", "M23perm", Join(["A", convert(i + 1, string), ":",
      "A", convert(i + 1, string)], ""))[1][1]) :
     if data12 > M12γ_calculated then n1 := n1 + 1 : end if:
     if data13 > M13γ_calculated then n2 := n2 + 1 : end if:
     if data23 > M23γ_calculated then n3 := n3 + 1 : end if:
  end do:
  P_{M12, calcXpermut} := evalf(n1/B, 3); P_{M13, calcXpermut} := evalf(n2/B, 3); P_{M23, calcXpermut}
      := evalf(n3/B, 3);
```

```
> # Reading files and calculation of P-values among matrices
  (bootstrap versus bootstrap)
```

> $n1 := 0 : n2 := 0 : n3 := 0 :$

for i **from** 1 **by** 1 **to** B **do**

$data12 := (Import("Results.xls", "M12", Join(["A", convert(i+1, string), ":", "A", convert(i+1, string)], ""))[1][1]) :$

$data13 := (Import("Results.xls", "M13", Join(["A", convert(i+1, string), ":", "A", convert(i+1, string)], ""))[1][1]) :$

$data23 := (Import("Results.xls", "M23", Join(["A", convert(i+1, string), ":", "A", convert(i+1, string)], ""))[1][1]) :$

if $data13 > M23\gamma_{bootstrap}$ **then** $n1 := n1 + 1$: **end if**:

if $data12 > M23\gamma_{bootstrap}$ **then** $n2 := n2 + 1$: **end if**:

if $data12 > M13\gamma_{bootstrap}$ **then** $n3 := n3 + 1$: **end if**:

end do:

$P_{M23,\,M13} := evalf\left(\frac{n1}{B}, 3\right); P_{M23,\,M12} := evalf\left(\frac{n2}{B}, 3\right); P_{M13,\,M12} := evalf\left(\frac{n3}{B}, 3\right);$

$$P_{M23,\,M13} := 0.$$

$$P_{M23,\,M12} := 0.$$

$$P_{M13,\,M12} := 0. \quad (1)$$

```
> # PART 8: READING AND COMPUTATION OF SIMILARITY
  MATRICES AND BINARY MAPS (DERMATOME SEQUENCE)
> # Loading packages...
> restart:
> with(ExcelTools):
  with(ListTools):
  with(StringTools):
  with(plots):
  with(ArrayTools):
  with(LinearAlgebra):
  interface(warnlevel = 0):
> acupoints := 361:
> # Reading files with matrices
> Lini := convert(2, string) : Lfim := convert(acupoints + 1, string) : C := ("P") : cell
      := Join([C, Lini, ":", C, Lfim], "") :
  DERM := (Import("Additional file 1.xls", "Acupoints", cell)) :
  M1 := ImportMatrix("M1data.txt", source = delimited, datatype = numeric) :
  M2 := ImportMatrix("M2data.txt", source = delimited, datatype = numeric) :
  M3 := ImportMatrix("M3data.txt", source = delimited, datatype = numeric) :
> # Filling the main diagonal
> for i from 1 by 1 to acupoints do
   for j from 1 by 1 to acupoints do
     if i = j then M1[i, j] := 1 : M2[i, j] := 1 : M3[i, j] := 1 : end if:
   end do:
   end do:
> # Analysis by dermatome level
> # Generating unique dermatome levels combinations
> LEVEL := convert(sort(MakeUnique(convert(DERM, list))), Array) :
  N := ArrayNumElems(LEVEL) :
> # "For loop" for sorting acupoints by first dermatome level
> rank := Array(1 ..acupoints) :
  freq := Array(1 ..N) :
> for i from 1 by 1 to acupoints do
    for j from 1 by 1 to N do
     if evalb(DERM[i][1] = LEVEL_j) = true then freq_j := freq_j + 1; rank_i := j else freq_j
       = freq_j; rank_i := rank_i : end if:
    end do:
  end do:
> rankM1 := Matrix(1 ..acupoints, 1 ..acupoints) :
  rankM2 := Matrix(1 ..acupoints, 1 ..acupoints) :
  rankM3 := Matrix(1 ..acupoints, 1 ..acupoints) :
  surrM1 := Matrix(1 ..acupoints, 1 ..acupoints) :
  surrM2 := Matrix(1 ..acupoints, 1 ..acupoints) :
  surrM3 := Matrix(1 ..acupoints, 1 ..acupoints) :
> rank1 := rank:
  for i from 1 by 1 to acupoints do
   for j from 1 by 1 to acupoints do
    rankM1_{i,j} := M1[(max(rank1[i], rank1[j])), (min(rank1[i], rank1[j]))];
    rankM2_{i,j} := M2[(max(rank1[i], rank1[j])), (min(rank1[i], rank1[j]))];
```

```
rankM3[i,j] := M3[(max(rank1[i], rank1[j])), (min(rank1[i], rank1[j]))];
  end do:
 end do:
> # Permutation test (surrogate analysis): Random ordering
  (shuffle of acupoints for comparison to the sorted matrices)
> rank2 := Statistics[Shuffle](rank) :
 for i from 1 by 1 to acupoints do
  for j from 1 by 1 to acupoints do
   surrM1[i,j] := M1[(max(rank2[i], rank2[j])), (min(rank2[i], rank2[j]))];
   surrM2[i,j] := M2[(max(rank2[i], rank2[j])), (min(rank2[i], rank2[j]))];
   surrM3[i,j] := M3[(max(rank2[i], rank2[j])), (min(rank2[i], rank2[j]))];
  end do:
 end do:
> # Plotting array of 2D-matrices
> AA := Array(1..2, 1..3) :
 AA[1,1] := matrixplot(rankM1, style = surface, shading = zgrayscale, title
    = ["Similarity on dermatomes\n"], labels = ["Acupoint", "Acupoint", ""],
    labeldirections = ["vertical", "horizontal", "vertical"], font = [ARIAL, 13, BOLD], axes
    = normal, orientation = [0, 0, 0]) :
 AA[1,2] := matrixplot(rankM2, style = surface, shading = zgrayscale, title
    = ["Similarity on traditional actions\n(dermatome sequence)"], labels = ["Acupoint",
    "Acupoint", ""], labeldirections = ["vertical", "horizontal", "vertical"], font = [ARIAL,
    13, BOLD], axes = normal, orientation = [0, 0, 0]) :
 AA[1,3] := matrixplot(rankM3, style = surface, shading = zgrayscale, title
    = ["Similarity on contemporary indications\n"], labels = ["Acupoint", "Acupoint", ""],
    labeldirections = ["vertical", "horizontal", "vertical"], font = [ARIAL, 13, BOLD], axes
    = normal, orientation = [0, 0, 0]) :
 AA[2,1] := matrixplot(surrM1, style = surface, shading = zgrayscale, title
    = ["Similarity on dermatomes\n"], labels = ["Acupoint", "Acupoint", ""],
    labeldirections = ["vertical", "horizontal", "vertical"], font = [ARIAL, 13, BOLD], axes
    = normal, orientation = [0, 0, 0]) :
 AA[2,2] := matrixplot(surrM2, style = surface, shading = zgrayscale, title
    = ["Similarity on traditional actions\n(after permutation)"], labels = ["Acupoint",
    "Acupoint", ""], labeldirections = ["vertical", "horizontal", "vertical"], font = [ARIAL,
    13, BOLD], axes = normal, orientation = [0, 0, 0]) :
 AA[2,3] := matrixplot(surrM3, style = surface, shading = zgrayscale, title
    = ["Similarity on contemporary indications\n"], labels = ["Acupoint", "Acupoint", ""],
    labeldirections = ["vertical", "horizontal", "vertical"], font = [ARIAL, 13, BOLD], axes
    = normal, orientation = [0, 0, 0]) :
> display(AA);
> # End of routine
```
